# Biomarkers of Periodontitis and Its Differential DNA Methylation and Gene Expression in Immune Cells: A Systematic Review

**DOI:** 10.3390/ijms231912042

**Published:** 2022-10-10

**Authors:** Angélica M. Cárdenas, Laura J. Ardila, Rolando Vernal, Samanta Melgar-Rodríguez, Hernán G. Hernández

**Affiliations:** 1 Faculty of Dentistry, Universidad Santo Tomás, Bucaramanga 680001, Colombia; 2 Doctoral Program in Dentistry, Faculty of Dentistry, Division of Health Sciences, Universidad Santo Tomás, Carrera 27 Floridablanca Highway 80-395, Bucaramanga 680001, Colombia; 3 Periodontal Biology Laboratory, Faculty of Dentistry, Universidad de Chile, Santiago 8380492, Chile; 4 Department of Conservative Dentistry, Faculty of Dentistry, Universidad de Chile, Santiago 8380492, Chile

**Keywords:** DNA methylation, gene expression, periodontitis, epigenomics, systemic biomarkers

## Abstract

The characteristic epigenetic profile of periodontitis found in peripheral leukocytes denotes its impact on systemic immunity. In fact, this profile not only stands for periodontitis as a low-grade inflammatory disease with systemic effects but also as an important source of potentially valuable clinical biomarkers of its systemic effects and susceptibility to other inflammatory conditions. Thus, we aimed to identify relevant genes tested as epigenetic systemic biomarkers in patients with periodontitis, based on the DNA methylation patterns and RNA expression profiles in peripheral immune cells. A detailed protocol was designed following the Preferred Reporting Items for Systematic Review and Meta-analysis -PRISMA guideline. Only cross-sectional and case-control studies that reported potential systemic biomarkers of periodontitis in peripheral immune cell types were included. DNA methylation was analyzed in leukocytes, and gene expression was in polymorphonuclear and mononuclear cells. Hypermethylation was found in TLR regulators genes: *MAP3K7*, *MYD88*, *IL6R*, *RIPK2*, *FADD*, *IRAK1BP1*, and *PPARA* in early stages of periodontitis, while advanced stages presented hypomethylation of these genes. *TGFB1I1*, *VNN1*, *HLADRB4*, and *CXCL8* genes were differentially expressed in lymphocytes and monocytes of subjects with poorly controlled diabetes mellitus, dyslipidemia, and periodontitis in comparison with controls. The *DAB2* gene was differentially overexpressed in periodontitis and dyslipidemia. Peripheral blood neutrophils in periodontitis showed differential expression in 163 genes. Periodontitis showed an increase in ceruloplasmin gene expression in polymorphonuclears in comparison with controls. Several genes highlight the role of the epigenetics of peripheral inflammatory cells in periodontitis that could be explored in blood as a source of biomarkers for routine testing.

## 1. Introduction

Periodontitis, nowadays termed as periodontitis stages III/IV according to the 2017 Classification of Periodontal and Peri-implant Diseases and Conditions [1], is considered the sixth most prevalent osteolytic disease in humans [2]. In fact, periodontitis affects nearly 11.2% of the world population and is strongly associated with systemic diseases being considered as a public health problem [2,3]. Pathologically, periodontitis is defined as an inflammatory disease caused by dysbiotic changes of the subgingival microbiota attached to the tooth, which leads to a deregulated osteolytic immune response and, finally, tooth loss [4,5,6]. In this context, the host’s immune response can be modified by both genetic and epigenetic factors that remodel chromatin, causing the activation or deactivation of genes that can determine a differential susceptibility to the development of periodontitis and other inflammatory comorbidities [7]. In general, evidence has shown that conserved epigenetic mechanisms contribute to the maintenance of inflammation and physiologic/aberrant states related to gene expression changes [8]. According to this view, DNA methylation is fundamental for differential gene expression because epigenetic modifications that vary between different cell types promote changes in gene expression, affecting their phenotype and behavior during disease/health states [9,10].

In this context, the role of epigenetic mechanisms, such as DNA methylation, during the progression of periodontitis and its association with systemic diseases have been described. For instance, hypomethylated states of the promoter regions of genes encoding pro-inflammatory molecules such as interleukin (IL)-6, IL-8, IL-10, INF-γ, and IL-17 have been identified in human gingival biopsies and associated to a possible response against periodontal bacteria [11,12,13]. In addition, gingival epithelial cells showed differential promoter-level hypermethylation of *IL12A*, *TLR2*, *GATA3*, and hypomethylation of *ZNF287* and *STAT5A* genes in response to stimulation with the periodontal pathogen *Porphyromonas gingivalis* (*P.g*) [14]. Similarly, a positive correlation has been found between *TLR-2* promoter hypermethylation in inflammatory cells from gingival tissue biopsies and increased pocket probing depth in periodontitis-affected patients [15]. Likewise, differentially methylated CpG sites have been identified at the *thioredoxin* gene in subjects with periodontitis, which expression is involved in the activation pathways of the innate immune response and, particularly, the activation of the IL-1β mediated response to pathogen-associated molecular patterns [16]. Such epigenetic modification has also been linked to insulin resistance, suggesting an interaction between periodontitis and diabetes [16].

Therefore, identifying epigenetic profiles and how they determine the expression of genes related to the periodontal immune response could be useful to identify potential risk markers of periodontitis onset and progression, and factors related to its comorbidities [17]. In fact, the use of epi-markers as prognostic, diagnostic, or therapeutic tools could be relevant in clinical decision-making for the application of personalized therapeutic programs [18]. Moreover, increased DNA methylation levels found in peripheral blood samples have been associated with Alzheimer’s disease [19,20], genetic risk in rheumatoid arthritis [21], and diabetic nephropathy [22]. Even though DNA methylation is a promising biomarker for many diseases [23], there are no available systematic reviews that summarize epigenetic changes in immune cell genes during periodontitis. Thus, the aim of this study is to identify relevant genes to be tested as epigenetic systemic biomarkers present in periodontitis patients based on the DNA methylation patterns/mRNA expression profiles in peripheral immune cells.

## 2. Materials and Methods

A detailed protocol was designed according to the PRISMA 2020 statement [24]. The process of study selection using PRISMA flow diagram for systematic reviews which included searches of databases, registers, and other sources, can be found at: https://prisma-statement.org//PRISMAStatement/FlowDiagram. Protocol registration can be found at PROSPERO (registration number CRD42021270817, registration date was 26 November 2021).

### 2.1. Eligibility Criteria

Publications that met the following PECO statement and eligibility criteria were included to answer the research question: which genes are relevant to be tested as epigenetic systemic biomarkers present in periodontitis patients based on differential DNA methylation and differential gene expression in peripheral immune cell types, compared to healthy patients?

P: Immune cells obtained from peripheral blood samples from periodontitis patients. 

E: Periodontitis.

C: Healthy non-periodontitis individuals. 

O: Genes/regions with differential DNA methylation or mRNA expression (including a difference in the percentage of methylation between groups or gene expression fold-change and the corresponding *p*-values). 

#### 2.1.1. Inclusion Criteria

- Studies reporting potential systemic biomarkers of periodontitis based on differential DNA methylation and differential gene expression in peripheral immune cell types.
- Cross-sectional and case-control studies published since 2006 and up to July 2021 in the English language were considered.

#### 2.1.2. Exclusion Criteria

Studies not accessible in full text, animal studies, and in vitro studies were excluded.

### 2.2. Information Sources and Search Strategy 

The electronic search was performed in Medline via PubMed, Web of Science, and Scopus databases (Table 1). Additionally, searching strategies on Gene Expression Omnibus and Google Scholar were made, using the corresponding adapted equations (Table 1).

### 2.3. Data Selection and Extraction

The selection and extraction processes were carried out by two independent authors (AMC/LJAE) who retrieved study records from databases into Microsoft Excel tables. Then, duplicates were removed, and selection criteria were applied. Articles were excluded by title and then by abstract, and finally by full-text revision to obtain the final selection. Then, a predesigned Excel sheet was separately fulfilled by AMC and LJAE with the selected variables for each included study. In case of discrepancy, the records were reviewed by the senior researchers (HGH/RV). Then, the following data were extracted from the included studies: Title, abstract, year, settings, dataset accessibility, GEO code, cell type and source, number of periodontitis cases analyzed, objectives, subject or population, comparison, number of healthy control individuals, molecular technique, genes evaluated, evaluation technique, main results of differentially expressed/methylated genes, gene ontologies found in gene enrichment analysis, authors’ conclusions, and conflicts of interest.

### 2.4. Outcome Measures 

To evaluate which genes are relevant to be tested as epigenetic systemic biomarkers present in periodontitis patients, the primary outcome measures were genes/regions with differential DNA methylation or mRNA expression. Otherwise, the ontological terms found in gene enrichment analysis of the differentially methylated or differentially expressed genes were considered secondary outcome measures.

### 2.5. Risk of Bias Assessment

The risk of bias (RoB) was assessed independently and in duplicate by two reviewers (LJAE and AMC), previously calibrated in RoB assessment rounds, with disagreements being resolved by consensus. The assessment was carried out using the Newcastle-Ottawa Scale (NOS) for case-control studies and a NOS-modified version for cross-sectional studies. Only cross-sectional studies were finally assessed.

### 2.6. Data Synthesis

The main results of the selected studies were extracted by identifying the genes of interest and their behavior in healthy individuals and periodontitis subjects. A comparison and association thereof were made between the different records using the datasheet. Following the stipulations of the PRISMA checklist, the data synthesis was performed by two researchers independently, and any discrepancies were revised by a senior researcher. Results were synthesized by grouping the studies by cell types (leukocytes, neutrophils, or monocytes) and types of extracted data (DNA methylation and gene expression).

## 3. Results

### 3.1. Data Selection

**Figure 1:**
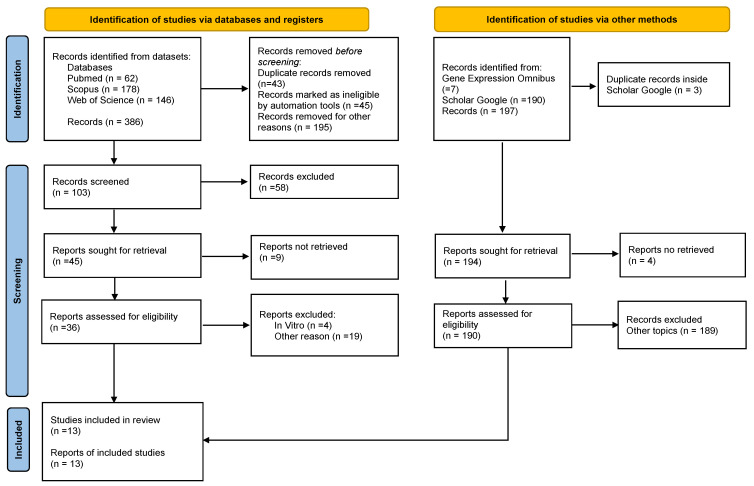
PRISMA 2020 Flow diagram depicting the selection of the eligible studies.

The electronic searches yielded 386 publications’ registers from the databases. Using the year exclusion criteria and eliminating duplicated articles, 283 articles were excluded, resulting in a total of 103 potentially relevant articles that were chosen for title and abstract evaluation. After screening 103 records, 58 were excluded. Then, forty-five articles were sought for retrieval and evaluation. After excluding reports that did not meet the inclusion criteria, 13 studies were finally included in the review. (Figure 1). No case-control studies were identified.

The process of study selection used PRISMA flow diagram for systematic reviews which included searches of databases, registers, and other sources from the PRISMA 2020 statement. Available at: https://prisma-statement.org//PRISMAStatement/FlowDiagram, registration date was 26 November 2021).

In addition, 190 articles were found on Google Scholar and from them, one article was included in the review. The search landed seven results on Gene Expression Omnibus, but none of them met the inclusion criteria.

### 3.2. Description of the Studies

Overall, the studies evaluated inflammatory mediator genes, with six studies analyzing the whole genome, three of them assessing several genes and one study assessing only one gene. Of the 13 included studies, both DNA methylation and gene expression were analyzed in only one study [25], only DNA methylation was analyzed in leukocyte cells in five studies [17,26,27,28,29], and two gene expression was analyzed in polymorphonuclear (PMNs) and mononuclear cells in five studies [30,31,32,33,34,35,36].

Based on the different cell types, six studies evaluated peripheral blood leukocytes [17,25,26,27,28,29] (Table 2), two evaluated PMNs or their subcomponents (neutrophils) [30,31] (Table 3), and five evaluated peripheral blood mononuclear cells (PBMCs) or their subcomponents (lymphocytes and monocytes) [32,33,34,35,36] (Table 4). All the included studies were cross-sectional. Four studies were conducted in North America (USA: 4), four studies in Europe (UK: 2, Denmark: 1, Germany: 1), three studies in South America (Brazil: 2, Colombia: 1), and two in Asia (Japan: 2) (Table 2, Table 3 and Table 4).

Most studies defined periodontitis according to the clinical parameters criteria of the 1999 classification of the American Association of Periodontology and one study used self-reported parameters associated with periodontitis. Patients affected by rheumatoid arthritis were included in two studies [28,29] and patients affected by type 2 diabetes mellitus were included in one study [35]. Only two studies included smokers. (Supplement Materials, Tables S1–S5).

### 3.3. Risk of Bias Assessment

According to the Newcastle-Ottawa scale, nine out of 13 studies had very good quality, four had good quality, and one study was evaluated as satisfactory (Table 5).

### 3.4. DNA Methylation in Peripheral Blood Leukocytes

DNA methylation in peripheral blood leukocytes (PBL) was evaluated in six studies [17,25,26,27,28,29]. (Table 2). Four of these studies used methods to detect changes in a single locus, such as: pyrosequencing [26], direct bisulfite sequencing [28,29], and methylation-specific PCR [27]. On the other hand, two studies were conducted using Illumina human DNA methylation array technology [17,25]. The first one used the 450K DNA methylation platform [25] and the study by Hernandez et al., used the most recent version of this technology: Illumina MethylationEPIC BeadChip [17] (Table 6).

Among the studies conducted in PBLs (Table 6), Shaddox et al., performed a DNA methylation analysis of the regulators of the TLR pathway in periodontitis in children and young adults. Early stages of periodontitis presented hypermethylation in CpGs compared with healthy controls in the promoter regions of the following TLR regulator genes: MAP3K7, MYD88, IL6R, RIPK2, FADD, IRAK1BP1, and PPARA, while advanced stages of periodontitis presented hypomethylation of these genes in comparison to early stages of periodontitis [26]. (Supplement Materials, Table S3).

In periodontitis, two studies evaluated methylation status in PBLs [28,29]. One of them assessed the DNA methylation of the TNF gene promoter region, demonstrating the hypermethylation of over 12 dinucleotides of CpG during periodontitis [28]. (Supplement Materials, Table S3). The study by Ishida et al., (2012) analyzed the methylation patterns of the IL6 promoter gene in peripheral blood leukocytes from periodontitis patients in comparison to healthy individuals and subjects affected by rheumatoid arthritis (RA). The IL6 promoter gene was found to contain 19 CpG motifs where CpG motif methylation levels at −74 bp from canonical Transcription Start Site (TSS) were in the hypomethylated state in individuals with periodontitis and arthritis in comparison to healthy controls (*p* = 0.0001) [29]. (Supplement Materials, Table S3). In another single locus study, a methylation analysis was performed on leukocyte blood cells from smokers with periodontitis, defined as “Subjects with at least three teeth exhibiting 5 mm CAL sites, in at least two different quadrants”, and significant differences in the DNA methylation levels of the IL8 promoter gene were not found compared to non-smokers with periodontitis [27] (Supplement Materials, Table S3). 

Using the Infinium 450 K DNA methylation platform, women with a self-reported diagnosis of periodontitis (who presented with positive dental traits for gingival bleeding and tooth mobility) showed hypomethylated states of the ZNF804A (cg21245277; beta = −0.33, *p*-value = 7.17 × 10^−8^, FDR = 0.03) and XKR6 genes (cg11051055; beta = −0.49, *p*-value = 1.53 × 10^−8^, FDR = 0.003), respectively. In addition, a hypermethylated state of the IQCE gene was observed in tooth mobility, at the cg08157914 site (beta = 0.38, *p*-value = 6.85 × 10^−8^, FDR < 0.001) [25]. It is important to highlight that the identification of genes with hypomethylated states in individuals with gingival bleeding would correspond to an increase in the upregulation of gene expression, so ZNF804A should be considered as a gene related to gingival tissue inflammation. (Supplement Materials, Table S3).

More recently, Hernández et al. used the Infinium EPIC DNA methylation platform to test differential methylation between periodontitis cases and healthy individuals [1] (Table 2B). Their results showed 81 differentially hypermethylated genes and 21 differentially hypomethylated genes in periodontitis subjects in comparison to periodontally healthy individuals. In particular, two genes differentially hypermethylated in periodontitis, ZNF718 and HOXA4, supported by several CpG sites for both analyses: differentially methylated positions and differentially methylated regions; similarly, the ZFP57 gene was differentially hypomethylated in both analyses in periodontitis patients [17]. (Supplement Materials, Table S3). The corresponding functional gene enrichment analysis showed a robust relation between the differentially methylated genes in periodontitis with the activation of the immune response against bacteria and the antigenic processing and presentation ontologies [17] (Table 6).

### 3.5. Gene Expression in Polymorphonuclears Cells

Two studies in this systematic review focused on the gene expression in polymorphonuclear cells in periodontitis [30,31] (Table 3). Wright et al., (2008) stated that the hyperinflammatory neutrophil phenotype associated with periodontal tissue damage could be defined by genetic alterations produced during the chronic inflammatory response [30]. Indeed, the gene expression signature analysis of peripheral blood neutrophils by means of genome-wide analysis with HG_U133A in subjects with periodontitis found differential expression in 163 genes (149 upregulated, 14 downregulated) in comparison to healthy individuals. Moreover, the gene expression analysis showed the upregulation of MX1, IFIT4, G1P2, IFIT1, CIG5, and IFI44, further corroborated by Reverse transcription PCR (RT-PCR) [30]. 

Iwata et al., (2009) found an overexpression of the ceruloplasmin (CP) gene in PMNs from periodontitis patients in comparison to healthy subjects by means of quantitative RT-PCR. CP is a ferroxidase enzyme that participates in the transport and metabolism of iron, the levels of iron ions increase during inflammation and hypoxia, which in turn increase the production of superoxide levels by PMNs [31] (Table 7) (Supplement Materials, Table S4).

### 3.6. Gene Expression in Peripheral Blood Mononuclear Cells (Lymphocytes and Monocytes) 

PBMCs, including lymphocytes and monocytes, are important cell types in periodontitis, which interact with periodontal bacteria and mediate host immune response [34]. Although the major damage occurs in periodontal tissues, peripheral blood leukocytes, as the source of local leukocytes, can contribute to periodontitis by influencing the destructive host immune response [32].

The study by Gonzales et al., (2012), was the only one specifically focused on CD4^+^ T cells, in which the expression of T helper type (Th)1 and Th2 cytokines (IL-2, IFN-γ, IL-4 and IL-13) was evaluated in peripheral blood samples from periodontitis patients. The results showed a decreased IL4 expression in periodontitis subjects in comparison with controls (17.8 ± 3.6 vs. 41.5 ± 2.1; *p* = 0.05). In addition, when evaluating inactivated CD4^+^ cells, the expression of IL4 (17.8 ± 3.6 Relative Fluorescence Unit [RFU]), IL13 (19.1 ± 2.8 RFU), and IL2 (18.6 ± 2.8 RFU) were higher than the expression of IFNG (4.9 ± 0.2 RFU) in the periodontitis group; otherwise, the expression of IL4 was greater than the expression of IL2 (10.5 ± 2.6) and IFNG (5.7 ± 1.8) in the cells of the healthy group. The authors stated that the increased IFNG expression in the cells of the healthy controls points out the key role of this cytokine in the regulation of the early immune response [33] (Table 8) (Supplement Materials, Table S5).

The other four studies have assessed PBMCs response to identify possible candidate genes associated with periodontitis and systemic diseases including type 2 diabetes mellitus (T2DM), dyslipidemia (DL), and rheumatoid arthritis (RA) [32,34,35,36] (Table 4). Sorensen et al., (2018) analyzed PBMCs from subjects diagnosed with untreated periodontitis, juvenile idiopathic arthritis (JIA), and rheumatoid arthritis using HG-U133A expression array to postulate common genes in general chronic inflammation and periodontitis [32].

Genome-wide analysis of gene expression showed that 53 transcripts were differentially expressed in periodontitis subjects in comparison to healthy controls, with 1 transcript (interleukin-32 [IL32]) being differentially decreased. Among them, MYOM2 and TLR2 genes were significantly upregulated in patients with periodontitis in comparison to healthy controls (*p* = 0.032 and *p* = 0.003, respectively), confirmed by RT-PCR. In addition, there were significantly overexpressed genes related to the immune responses (14 genes), response to external stimulus (16 genes), apoptosis (12 genes), cytokine activity (five genes), and chemotaxis (six genes) in samples from patients with periodontitis [32] (Table 8). 

Later, by using the RNA-seq approach, the transcriptome of mononuclear cells was analyzed in patients with periodontitis without comorbidities. The results showed 380 differentially expressed transcripts in periodontitis in comparison to controls (posterior probability of equal expression < 0.05 and posterior probability of differential expression > 0.95). Specifically, there were 228 and 152 transcripts upregulated and downregulated in periodontitis (corresponding to a total of 5955 isoforms). Among these results, some genes associated with periodontitis compared to microarray dataset, FACR, and CUX1. DAVID analysis showed that several genes were related to important biological activities in periodontitis, such as: endocytosis, cytokine production, and apoptosis [34] (Table 8) (Supplement Materials, Table S5).

In the study by Corbi et al., (2020), PBMCs were analyzed by microarray using Affymetrix and RT-qPCR to validate the differentially expressed genes (DEGs) in patients affected by periodontitis alone or associated with dyslipidemia (DL) and type II diabetes mellitus (T2DM), in comparison to healthy subjects (HS) (Table 8). The results showed that the circulating lymphocytes and monocytes of patients with periodontitis, T2DM, and DL exhibited a deregulated molecular profile, as follows: in subjects with poorly controlled T2DM versus HS: 1374 upregulated and downregulated DEGs. In subjects with well-controlled T2DM versus HS: 869 DEGs. In subjects with periodontitis plus DL versus HS: 521 upregulated and downregulated DEGs, and in subjects with periodontitis versus HS: 564 upregulated and downregulated DEGs. The validation analyses using RT-qPCR showed that four genes were differentially expressed in subjects with poorly controlled T2DM plus DL plus periodontitis compared to HS (TGFB1I1, VNN1, HLADRB4 and CXCL8). In subjects with DL plus periodontitis versus HS, three DEG were confirmed (DAB2, CD47, and HLADRB4), and in the comparison between subjects with periodontitis alone versus HS: the IGHG3 gen (IGHDL-P) was upregulated, and the ITGB2 and HLADRB4 genes were downregulated. In subjects with well-controlled T2DM plus DL plus periodontitis versus HS, there were 3 DEG genes (BPTF, PDE3B, and FN1) [35] (Table 8). (Supplement Materials, Table S5).

Another study evaluating PBMCs assessed the gene expression of the TLR pathway genes and miRNA regulators related to pathogenic mechanisms from subjects diagnosed with periodontitis. Gonçalves et al., (2022) evaluated 84 genes and 84 miRNA genes by PCR Arrays and miScript PCR Arrays, respectively. Among the 84 genes of the TLR pathway, 5 genes were upregulated in periodontitis patients compared to healthy controls: TLR2, TICAM-1 (TRIF), IRAK1, FOS, and CCL2. Among the 84 miRNAs, 8 presented fold-change > 2 in subjects with periodontitis in comparison to controls; 6 of such genes were found significantly upregulated in subjects with periodontitis in comparison to controls in a subsequent RT-PCR assay: MIR9-1, MIR155, MIR203A, MIR147A, MIR182, MIR183 genes [36] (Table 8) (Supplement Materials, Table S5).

## 4. Discussion

This is the first systematic review focused on the identification of relevant genes to be tested as systemic-blood biomarkers present in periodontitis patients based on studies of DNA methylation patterns and/or RNA expression profiles in peripheral immune cell types regarding their particular functions (Supplement Materials, Table S6). All the available studies in this review were of cross-sectional design, and all of them presented a favorable RoB evaluation. 

Understanding epigenetic mechanisms such as DNA methylation in the context of differential gene expression is relevant to the pathogenesis of immunoinflammatory diseases, such as periodontitis. Specifically, DNA methylation modifications can modulate gene expression and provoke alterations in cellular functioning at the local and systemic level, vary between different cell types, and favor the risk of appearance and/or progression of different diseases [9,10]. For example, the pioneer results shown by Ishida et al., (2012), where methylation patterns of the *IL6* promoter gene was differentially hypomethylated in an individual with periodontitis and rheumatoid arthritis, could indicate that the hypomethylated state of a single CpG in the *IL6* promoter region may promote higher serum levels of *IL6*, supporting an important role for this cytokine in the pathogenesis of chronic inflammatory diseases such as rheumatoid arthritis and periodontitis [29].

In this review, we have found and summarized common gene differential expressions identified by multiple studies. Among them, the *DAB2* gene was differentially overexpressed in two studies: Y.-Z. et al., (2016), in periodontitis, and Corbi S.C.T. et al., (2020), in periodontitis combined with dyslipidemia. In turn, Corbi et al., (2020) found that *HLADRB4* was downregulated in periodontitis alone, but interestingly, also in the combination of periodontitis with dyslipidemia and diabetes, being consistently detectable in circulating lymphocytes and monocytes [35]. Additionally, it showed that lymphocytes and monocytes express a dysregulated inflammatory profile in patients with periodontitis and systemic diseases [35]. On the other hand, *MYD88* was found to be differentially hypermethylated in leucocytes of patients with periodontitis [26] but differentially over-regulated in the transcriptome of PBMCs [34]. These discordant results could be explained by the different cell types evaluated by the authors.

In the studies reviewed in this work, the *IRAK1* gene was reported by Gonçalves-Fernandes et al., (2020) as being upregulated in patients with periodontitis [36]. The *IRAK1* gene encodes interleukin-1 receptor-associated kinase 1 and is related to IL1-mediated upregulation of NF-κβ. Interestingly, Oseni et al., have recently reported that DNA methylation regulates *IRAK1* expression in inflammatory contexts [37,38]. 

Remarkably, Shaddox et al., (2017), reported a differential hypomethylation in three CpG positions of *RIPK2* in patients with advanced stages of periodontitis, but the opposite result was found, in patients with early stages of periodontitis (CpGs 2, 3, and 5). These results may be related to the fact that *RIPK2* constitutes a marker of periodontitis detectable in peripheral blood, which could be used to differentiate such phenotypes. The same contrary and distinctive direction from the healthy controls for moderate or severe periodontitis was found in one position for *PPARA* (CpG 2) and for *MAP3K7* (primers *MAP3K7*-02:CpG 3) [26]. Additionally, Shaddox et al., (2017) found hypermethylation or hypomethylation detectable in TLR up-regulator and TLR down-regulator genes, indicating that the TLR signaling pathway could be modulated in both senses, inducing, or delaying the advance of periodontitis depending on the severity of the disease [26].

The results presented here denote an increasing interest in establishing peripheral/systemic biomarkers to enhance precision/personalized periodontal medicine for diagnosis, treatment-response prediction, prognosis, and epigenetic treatment. DNA methylation is the major epigenetic mechanism associated with activating or inhibiting gene expression. This mechanism can change from cell to cell or inside the cell, and could favor the maintenance of inflammation [27]. It is noted that two studies included smokers, and the other two included diabetes patients (Table 1, Table 2 and Table 3), which could make periodontitis markers certainly different in the presence or absence of these important comorbidities. Therefore, it would be ideal to conduct future studies on the epigenetic profiles of diabetes and smoking, and how could they mutually impact the course of periodontitis. In the same way, the interpretation of the results in peripheral blood was consistently reflective of the systemic state of immune disruption, which would be important in this context and in relation to its potential utility as a biomarker.

Neutrophils are the first line of cellular defense in the periodontium and are recognized for developing a hyperinflammatory phenotype due to the chronic release of inflammatory mediators and ROS in response to bacterial challenges. Wright et al., (2008) and Iwata et al., (2009) highlight a key role of gene expression in neutrophils of patients with periodontitis, showing the upregulation of *MX1*, *IFIT4*, *G1P2*, *IFIT1*, *CIG5*, and *IFI44* and ceruloplasmin genes [30,31]. These results suggest a role in the generation of oxidative stress at the local level due to an increase in the conversion of iron ions mediated by *CP* expression and, also, the *IFN 1*-stimulated gene regulation could be a key determinant of the molecular phenotype of peripheral blood neutrophils in patients with periodontitis, favoring of periodontal tissue damage [30,31].

Although we identified several important systemic markers that could be postulated as prognostic and therapeutic targets in patients with periodontitis and comorbidities, such as diabetes and rheumatoid arthritis, there are obvious limitations in this report. First, this work has a limited number of studies and presents diverse ethnical differences among the studied populations that can result in large differences in characteristic methylation patterns. On the other hand, most of them have small sample sizes, and heterogeneity between studies was found regarding the methodologies used for methylation and gene expression analyses. None of the studies compared the methylation and gene expression patterns in the different degrees of severity of periodontal disease (i.e., gingivitis and different degrees of severity of periodontitis), nor other risk factors or indicators that could modulate the methylation and expression profile of each participant and in different types of cells. However, this review is the first one centered on transcriptional/epigenetic potential biomarkers in the inflammatory cells present in the peripheral blood of patients with periodontitis, considering appropriately the cell type of each finding.

Otherwise, periodontitis has been associated with systemic inflammation, which favors the occurrence and progression of diseases such as metabolic syndrome, cardiovascular diseases, cancer and neurodegenerative diseases [26,27,28]. Some genes differentially expressed in other diseases coincide with genes found in this review, e.g., *ZNF718* gene was found to be differentially hypermethylated in peripheral blood samples of asthma patients [39] and the promoter region of this gene was found to be differentially hypermethylated with an increase in the sex hormone-binding globulin (*SHBG*), a hormone associated to metabolic diseases (e.g., diabetes) [40,41]. Moreover, *TNF-α* is involved in biological processes, including cell proliferation, differentiation, apoptosis, lipid metabolism, and coagulation in cancer [42]. *IL6* gene plays an important role in oncogenesis, metastasis through downregulation of Cadherin 1, and apoptosis [42].

*ZNF804A*, *XKR6*, and *IQCE* genes have also been linked to other diseases, such as bipolar disorder, schizophrenia, memory loss, longevity, blood metabolite levels, asthma, and allergic rhinitis [43,44].

For future research, studies should include the reporting of lifestyle- and environment-related factors that may contribute to the development of epigenetic alterations at the cellular and tissue level, and that may also identify reproducible epi-markers that reveal the degree of susceptibility to the progression of the disease and contribute to the strengthening of personalized and accurate therapies for periodontitis. In addition, future studies of epigenetic biomarkers at the cell-type level in chronic inflammatory diseases are necessary to detect precise changes between individuals. This would reduce the prevalence of the disease, make clinical intervention less invasive, and reduce treatment costs. Validation studies would also be needed to determine the potential use of these peripheral blood biomarkers as risk identifiers and monitors. Despite these findings, more studies are still needed to understand and highlight the importance of epigenetic modifications and their effect on gene expression, and how they contribute to the deterioration of periodontal tissues and the severity of systemic diseases. The phenotypic traits of each cell type and their different responses to inflammatory processes should also be considered. 

## 5. Conclusions

Systemic epi-markers with epigenetic therapeutic potential were identified in periodontitis patients based on studies of DNA methylation patterns with RNA expression profiles in PBMCs, particularly lymphocytes and monocytes. These results highlight new therapeutic targets with diagnostic, prognostic, and therapeutic potential not only for subjects with periodontitis but also for those with other diseases such as diabetes and rheumatoid arthritis.

## Figures and Tables

**Table 1:** Databases and search strategies.

Database	Keywords
Medline via PubMed	(“Periodontitis” [Mesh]) AND (“Humans” [Mesh]) AND (“Gene Expression” [Mesh] OR “Methylation” [Mesh] OR “DNA Methylation” [Mesh] OR “Transcriptome” [Mesh] OR “Oligonucleotide Array Sequence Analysis” [Mesh] OR “Sequence Analysis, DNA” [Mesh]) AND (“Neutrophils” [Mesh] OR “Leukocytes” [Mesh] OR “Blood Cells” [Mesh] OR “Leukocytes, Mononuclear” [Mesh] OR “Monocytes” [Mesh] OR “Granulocytes” [Mesh] OR “Eosinophils” [Mesh] OR “Lymphocytes” [Mesh] OR “B-Lymphocytes” [Mesh] OR “T-Lymphocytes” [Mesh])
Web of Science	TS = (periodontitis AND “Gene Expression” OR “Methylation” OR “DNA Methylation” OR “Transcriptome” OR “Oligonucleotide Array Sequence Analysis” OR “Sequence Analysis, DNA” AND “Neutrophils” OR “Leukocytes” OR “Blood Cells”) TS = humans TI = periodontitis
Scopus	TITLE (periodontitis) AND humans ANDTITLE-ABSKEY (“Gene Expression” OR “Methylation” OR “DNA Methylation” OR “Transcriptome” OR “Oligonucleotide Array Sequence Analysis” OR “Sequence Analysis, DNA”) AND TITLE-ABS-KEY (“Neutrophils” OR “Leukocytes” OR “Blood Cells” OR “mononuclear Cells” OR monocytes OR granulocytes OR eosinophils OR lymphocytes OR “B cells” OR “T cells”)
Google Scholar	(intitle:”periodontal” OR intitle:”periodontitis”) AND (“gene expression” OR “DNA methylation” OR “transcriptome”) AND (“polymorphonuclear” OR “blood” OR “peripheral blood” OR “leucocytes” OR monocytes) AND (microarrays OR “microchip”)
Gene Expression Omnibus	periodontitis AND (“Gene Expression” OR “Methylation”) AND(“Neutrophils” OR “Leukocytes” OR “Blood Cells”)

**Table 2:** Studies assessing peripheral blood leucocytes cells (PBLCs) differential DNA methylation and gene expression.

Study/Year	Focus	Type of Study	Evaluated Genes	Nominal Condition(s) of Interest/Periodontitis Definition Criteria (Clinical Parameters)
Oliveira N.F.P. et al., 2009 [27]	DNA methylation status in the gene promoter of *IL8* in blood leucocytes	Cross-sectional	*IL8*	Periodontitis/At least three teeth exhibiting sites ≥ 5 mm CAL, in at least two different quadrants
Ishida K. et al., 2012 [29]	DNA methylation IL6 promoter in mononuclear cells	Cross-sectional	* IL6 *	Periodontitis or Rheumatoid Arthritis/Sites with probing depth (PD) ≥ 4 mm
Kojima A. et al., 2016 [28]	DNA methylation pattern of the TNF promoter in blood cells	Cross-sectional	*TNF*	Periodontitis or Rheumatoid Arthritis/Sites with probing depth (PD) ≥ 4 mm
Shaddox L.M. et al., 2017 [26]	DNA methylation promoter regions of genes involved in TLR in peripheral blood cells	Cross-sectional	*CD14*, *FADD*, *HRAS*, *HSPA1A*, *HSPD1*, *IL6R*, *IRAK1*, *IRAK2*, *IRF1*, *IRF3*, *IRF8*, *MAP3K7*, *MYD88*, *PPARA*, *RIPK2*, *TBK1*, *TLR2*, *TLR5*, *TOLLIP*, *TRAF6*, *UBE2N*, *UBE2V1*, *EP_SEC*, *EP_DEC* Selected after the screening: *FADD*, *MAP3K7*, *MYD88*, *PPARA*, *IRAK1*, *RIPK2*, *and IL6R*	Periodontitis/CAL ≥ 4 mm localized in at least two teeth (first molar)
Kurushima Y. et al., 2019 [25]	Epigenomic variation in peripheral whole blood using a twofold approach	Cross-sectional	Genome-wide analysis Loci-focused analysis: *NIN*, *ABHD12B*, *WHAMM*, *KCNK1*, *DAB2IP*, *CLEC19A*, *TRA*, *TM9SF2P*, *GGTA2P*, *IFI16*, *RBMS3*, *C1QTNF7*, *TSNARE*, *HPVC1*, *SLC15A4*, *PKP2*, *SNRPN*, *IL8*, *CD44*, *CXCL1*, *IL6ST*, *CCR1*, *MMP7*, *MMP13*, *MMP3*, *TLR9*, *IL18*, *IFNB1*, *GLT6D1*, *IL1B*, *IL1RN*, *IL6*, *IL10*, *VDR*, CD14, TLR4, MMP1 RNA-sequencing *ZNF804A*, *VDR*, *IL6ST*, *TMCO6*, *IL1RN*, *CD44*, *IL1B*, *WHAMM*, and *CXCL1*	Self-reported periodontitis traits/ • “Have you ever had the condition of gum bleeding” • “Have you ever had the condition of gum decay or loose teeth”
Hernández H.G. et al., 2021 [17]	DNA methylation in peripheral leukocytes	Cross-sectional	Genome-wide analysis	Periodontitis/NR

TLR, Toll-like receptors; NR, not reported; CAL, Clinical attachment loss.

**Table 3:** Studies assessing polymorphonuclears (PMNs) cells or subcomponents (neutrophils) differential gene expression.

Author/Year	Focus	Type of Study	Evaluated Genes	Nominal Condition(s) of Interest/Periodontitis Definition Criteria
Wright H.J. et al., 2008 [30]	To analyze the gene expression signature of hyperresponsive peripheral blood neutrophils from periodontitis patients	Cross-sectional	Genome-wide analysis	Periodontitis/At least two non-adjacent sites per quadrant exhibiting PPD ≥ 5 mm, with bleeding on probing, radiographic bone loss ≥ 30% and were not first molar or incisor sites.
Iwata T. et al., 2009 [31]	To evaluate ceruloplasmin expression and regulation in human PMNs from healthy donors and patients diagnosed with P.	Cross-sectional	* CP *	Periodontitis/CAL ≥ 4 mm localized in at least two teeth (first molar)

PMNs, polymorphonuclears; *CP*, Ceruloplasmin; P, Periodontitis; PPD, Probing pocket depths; CAL, Clinical attachment loss.

**Table 4:** Studies assessing Peripheral blood mononuclear cells (PBMCs) or subcomponents (lymphocytes and monocytes) differential gene expression.

Author/Year	Focus	Type of Study	Evaluated Genes	Nominal Condition(s) of Interest/Periodontitis Definition Criteria
Sørensen L.K. et al., 2008 [32]	Differentially expressed candidate genes in PBMCs	Cross-sectional	Genome-wide analysis	Periodontitis/CAL ≥ 4 mm localized at least two teeth or CAL ≥ 4 mm at least three teeth.
Gonzales J.R. et al., 2012 [33]	Expression and production of IL-2, IFNG, IL-4 and IL-13 in CD4^+^ cells from peripheral blood	Cross-sectional	Th1 and Th2 cytokines (*IL2*, *IFNG*, *IL4* and *IL13*)	Periodontitis/PPD and CAL ≥ 5 mm on at least one interproximal site affecting at least three teeth other than the first molars and incisors.
Liu Y.-Z. et al., 2016 [34]	Functional genes and pathways at monocyte transcriptomic level.	Cross-sectional	Genome-wide analysis	Periodontitis/CAL ≥ 5 mm
Corbi S.C.T. et al., 2020 [35]	Gene expression signatures from circulating lymphocytes	Cross-sectional	Genome-wide analysis	Periodontitis alone or associated with dyslipidemia and diabetes mellitus type 2/PD ≥ 6 mm and CAL ≥ 4 mm in at least 4 non-adjacent teeth.
Gonçalves Fernandes J et al., 2020 [36]	Gene expression of key TLR pathway genes and miRNA regulators in unstimulated PBMCs	Cross-sectional	84 genes from TLR pathway RT^2^ Profiler PCR Arrays 84 genes miRNA genes from miScript PCR Arrays Human Immunopathology	Periodontitis/At least 2 sites with CAL > 2 mm and radiographic bone loss on first molar or incisor.

PBMCs, peripheral blood mononuclear cells; TLR, Toll-like receptors; Th, T helper cells; PD, periodontal depth; CAL, clinical attachment loss.

**Table 5:** Newcastle-Ottawa scale assessment of cross-sectional studies.

Cross-Sectional Studies	Selection				Comparability	Outcomes		Total	Overall Quality Assessment
	Representativeness of the Sample	Sample Size	Non Respondents	Ascertainment of the Exposure		Assessment of Outcomes	Statistical Test		
Corbi S.C.T. et al., 2020	✯	✯	-	✯✯	✯✯	✯✯	✯	9✯	Very Good
Kojima A. et al., 2016	✯	✯	-	✯✯	✯✯	✯✯	✯	9✯	Very Good
Oliveira N.F.P. et al., 2009	✯	✯	-	✯✯	✯✯	✯✯	✯	9✯	Very Good
Sørensen L.K. et al., 2008	✯	✯	-	✯✯	✯✯	✯✯	✯	9✯	Very Good
Kurushima Y. et al., 2019	✯	✯	-	✯✯	✯✯	✯✯	-	8✯	Good
Hernández H.G. et al., 2021	✯	-	-	✯✯	✯✯	✯✯	✯	8✯	Good
Gonçalves Fernandes J et al., 2020	✯	✯	-	✯✯	✯	✯✯	✯	8✯	Good
Ishida K et al., 2012	✯	✯	-	✯✯	✯	✯✯	✯	8✯	Good
Wright HJ et al., 2008	✯	✯	-	✯✯	✯	✯✯	✯	8✯	Good
Shaddox L.M. et al., 2017	✯	✯	-	✯	✯	✯✯	✯	7✯	Good
Liu Y.-Z. et al., 2016	✯	✯	-	✯	✯	✯✯	✯	7✯	Good
Gonzales J.R. et al., 2012	✯	✯	-	✯	✯	✯✯	✯	7✯	Good
Iwata T et al., 2009	✯	✯	-	-	✯	✯✯	✯	6✯	Satisfactory

Good Studies: 7–8 points, Satisfactory Studies: 5–6 points, Unsatisfactory Studies: 0 to 4 points.

**Table 6:** DNA methylation and mRNA expression assessment in Peripheral Blood Leucocytes (PBL) studies.

Authors	Subject/ Population	Comparison	Expression Technique	Methylation Technique	Systemic Biomarkers Meth/mRNA
Oliveira N.F.P. et al., 2009 [27]	13 smokers with periodontitis 13 non-smokers with periodontitis	13 healthy (never smoked) control subjects (absence of CAL and no sites with probing depth > 3 mm)	NR	Methylation-specific PCR (MSP)	No DNA methylation/ Transcriptional expression biomarkers found for *IL8*
Ishida K et al., 2012 [29]	30 patients with RA and 30 patients with Periodontitis	30 age-, sex-, and smoking status–balanced healthy controls	NR	Direct bisulfite sequencing	Hypometh *IL6*
Kojima A. et al., 2016 [28]	30 patients with periodontitis (only) 30 patients with RA/ Japanese adults	30 race-matched healthy controls	NR	Direct bisulfite sequencing (Signal correction using ESME)	Hypermeth *TNF*
Shaddox L.M. et al., 2017 [26]	20 periodontitis/ African American 5–25 years old (10 initial and 10 advanced stages of the disease)	20 healthy unrelated controls	NR	EpiTect Methyl II PCR Array Human Toll-Like Receptor Signaling Pathway Signature Panel (Pyrosequencing)	Early stages of the disease Hypermeth *MAP3K7* Hypermeth *MYD88* Hypermeth *IL6R* Hypermeth *RIPK2* Hypermeth *IRAK1BP1* Hypermeth *PPARA* Hypermeth *FADD* Advanced stages of the disease Hypometh *RIPK2* Hypometh *MAP3K7* (at positions 1 and 3) Hypometh *MYD88* (at positions 1 and 5) Hypometh *IRAK1BP1* (at positions 1 and 3) Hypometh *PPARA* (at position 2)
Kurushima Y, et al., 2019 [25]	Patients with self-reported periodontitis Positive gingival bleeding trait: 269 female subjects (from 528 female individuals) Positive for tooth mobility trait: 121 female subjects (from 492 female individuals) RNA-sequencing (384 subjects) - Positive gingival bleeding trait: 342 (from 384 female individuals) - Positive for tooth mobility trait: 335 (from 384 female individuals)	Subjects without self-reported periodontitis Positive gingival bleeding trait: 259 female subjects (from 528 female individuals) Positive for tooth mobility trait: 371 female subjects (from 492 female individuals) RNA-sequencing (384 subjects) Negative gingival bleeding trait: 42 (from 384 female individuals) Negative for tooth mobility trait: 49 (from 384 female individuals)	RNA-sequencing	The Infinium Human Methylation 450 BeadChip	DNA Methylation in blood Hypometh *ZNF804A* ^‡^ in gingival bleeding. Hypermeth *IQCE* in tooth mobility Hypometh *XKR6* in tooth mobility *VDR*, *IL6ST*, *TMCO6*, *IL1RN*, *CD44*, *IL1B*, *WHAMM*, *and CXCL1* ^†^ mRNA Expression in Blood ↑ mRNA *ZNF804A*^‡^ in gingival bleeding *WHAMM*, *TMCO6* ^†^
Hernández H.G. et al., 2021 [17]	8 periodontitis patients	8 periodontally healthy subjects	NR	Illumina MethylationEPIC BeadChip (IMEB)	Hypermeth *ZNF718* Hypermeth *HOXA4* Hypometh *ZFP57*

Meth, methylation; ESME, Sanger/epigenetic sequencing methylation analysis; NR, not reported; CAL, clinical attachment loss; RA, Rheumatoid arthritis; Hypermeth, Differentially Hypermethylated; Hypometh, Differentially Hypomethylated; ↑ denotes increase in mRNA expression. **^†^** The direction of methylation difference was not reported. **^‡^** Correspondence of DNA methylation with gene expression for ZNF804A.

**Table 7:** Studies on mRNA expression in peripheral blood Polymorphonuclears (PMNs) or subcomponents (neutrophils).

Authors	Subject/ Population	Comparison	Cell Type(s)/ Source	Expression Analysis	Systemic Biomarkers mRNA
Wright HJ et al., 2008 [30]	19 patients with periodontitis (19, 36–61 years) (baseline and 3 months after periodontal treatment)	19 Age- and gender-matched periodontally healthy control subjects (37–62 years)	Neutrophils (discontinuous Percoll gradient isolation)/ peripheral blood	HG_U133A microarrays (Affymetrix) semi-quantitative RT-PCR	↑ mRNA *MX1*, *IFIT4*, *G1P2*, *IFIT1*, *CIG5*, and *IFI44-like*
Iwata T et al., 2009 [31]	36 patients with periodontitis (age range: 16 to 41 years)	36 systemically healthy control subjects (n = 36; age range: 21 to 39 years)	PMNs discontinuous gradient	RT-qPCR	↑ mRNA *CP*

HG, Human genome; CP, ceruloplasmin. Green arrow ↑ denotes increase in mRNA expression.

**Table 8:** Studies on mRNA expression in peripheral blood mononuclear cells (PBMCs) or subcomponents (lymphocytes and monocytes).

Authors	Subject/ Population	Comparison	Cell Type(s)/ Source	Expression Technique	Systemic Biomarkers mRNA
Sørensen L.K. et al., 2008 [32]	For microarray 5 Subjects with periodontally untreated: For RT-PCR 45 subjects with periodontitis	For microarray 2 controls with healthy periodontium (no interproximal attachment loss and no clinical signs of oral inflammatory conditions) For RT-PCR 25 healthy control subjects	Mononuclear cells (density centrifugation)/ peripheral blood	HG-U133A expression array RT-PCR, qPCR	Periodontitis ↑ mRNA *MYOM2* ↑ mRNA *TLR2*
Gonzales J.R. et al., 2012 [33]	20 periodontitis	20 non-periodontitis control subjects	CD4^+^ cells/peripheral blood	Real-time polymerase chain reaction (RT PCR) (TaqMan^®^)	In the inactivated CD4^+^ cells in periodontitis: ↓ mRNA *IL4*
Liu Y.-Z. et al., 2016 [34]	5 periodontitis subjects (non-smoking)	5 periodontally healthy	Mononuclear cells/peripheral blood	RNA-seq (Illumina TruSeq) microarray dataset GSE6751	Periodontitis pathogenesis ↑ mRNA *FACR*, *CLCN5 * *CUX1*, *RNASE3*, *REL*, *VNN2*, *SGMS2*, *GGT1*, *HLA-DOA*, *ME1* ↓ mRNA PXN-*AS1*, *URGCP*, *RPS20*, *FAM98A*, *XBP1*, *G3BP1*, *NFAT5* and *ZNF207.* Endocytosis ↑ mRNA *DENND1A*, *RUFY1*, *CORO1C*, *ASGR2*, *APP*, *DAB2*, *PICALM*, *CD36*, *AP1S2*, *CLEC7A*, *THBS1*, *CLCN5* and *RIN3 * Cytokine production ↑ mRNA *NLRC4*, *G6PD*, *MYD88*, *TLR4*, *NLRP3* and *PTAFR* Apoptosis ↑ mRNA *ARHGEF2*, *SGK1*, *DNM1L*, *XIAP*, *UBE4B*, *CIDEB*, *STK17B*, *TRIO*, *NLRP3*, *BCL2L13*, *NCSTN*, *TNFRSF1A*, *PEA15*, *NLRC4*, *APP*, *GSN*, *HIPK3*, *BNIP3L*, *NLRP12*, and *THBS1*
Corbi S.C.T. et al., 2020 [35]	24 periodontitis patients for Microarray expression (U133 Plus 2.0, Affimetrix): • 5 poorly controlled T2DM + dyslipidemia + periodontitis (T2DMpoorly-DL-P) • 7 well-controlled T2DM with dyslipidemia and periodontitis (T2DMwell-DL-P) • 6 well-controlled T2DM + dyslipidemia + periodontitis (DL-P) • 6 normoglycemic individuals + dyslipidemia + periodontitis (P) 120 periodontitis patients for RT-qPCR validation of selected DEGs: • 30 T2DMpoorly-DL-P • 30 T2DMwell-DL-P • 30 DL-P • 30 P	For Microarray U133 Plus 2.0: • 6 systemically healthy individuals without periodontitis (H) (homogeneity regarding biochemical, lipid and clinical periodontal parameters). For RT-qPCR validation: - 30 H	Mono-nuclear cells (Lymphocytes and monocytes)/peripheral blood	Expression Microarray U133 Plus 2.0 RT-qPCR (validation)	• P ↓ mRNA *IGHG3* ↑ mRNA *ITGB2* and *HLADRB4.* • T2DMpoorly + DL + P ↑ mRNA *TGFB1I1*, *VNN1* ↓ mRNA *HLADRB4* and *CXCL8* • T2Dmwell + DL + P ↓ mRNA *BPTF*, *PDE3B* ↑ mRNA *FN1* • DL + P ↑ mRNA *DAB2* ↓ mRNA*CD47* and *HLADRB4*
Gonçalves Fernandes J et al., 2020 [36]	For array screening. 10 subjects with periodontitis for mRNA screening RT2 Profiler PCR Arrays (TLR pathway) microRNA ARRAY • 11 subjects with periodontitis for microRNA screening by miScript Immunopathology PCR arrays (Qiagen) For gene expression validation: • 29 periodontally healthy subjects for mRNA(qPCR) • 31 periodontally healthy subjects for microRNAs (qPCR) African American subjects	For array screening. • 9 control subjects for mRNA screening RT^2^ Profiler PCR Arrays (TLR pathway) • 11 control subjects for microRNA screening by miScript Immunopathology PCR arrays (Qiagen) For gene expression validation: 29 periodontally healthy subjects for mRNA(qPCR) 31 periodontally healthy subjects for microRNAs (qPCR)	Mononuclear cells (SepMate™ Isolation method)/peripheral blood	-RT^2^ Profiler PCR Arrays (TLR pathway) -miScript PCR Arrays Human Immunopathology -RT-qPCR (validation)	↑ mRNA *TLR2*, *TICAM-1 (TRIF)*, *IRAK1*, *FOS*, *CCL2* ↑ mRNA *miRNAs MIR9-1*, *MIR155*, *MIR203A*, *MIR147A*, *MIR182*, *MIR183*

HG, Human genome; Red arrow ↓ and green arrow ↑ denote increase or decrease in mRNA expression; T2DM, Type 2 diabetes mellitus; P, periodontitis; DL, dyslipidemia.

## Data Availability

Not applicable.

## Supplementary Materials

The following supporting information can be downloaded at: https://www.mdpi.com/article/10.3390/ijms231912042/s1.
