# Supplementary material for: Biomarkers of Periodontitis and Its Differential DNA Methylation and Gene Expression in Immune Cells: A Systematic Review
Source: Int J Mol Sci. 2022 Oct 10;23(19):12042. doi: 10.3390/ijms231912042 (PMC9570497; doi:10.3390/ijms231912042)
Supplement: Supplementary file 1 [file ijms-23-12042-s001.zip › Tabla S1.pdf]

Table S1. General characteristics of studies assessing peripheral blood leucocytes cells (PBLCs) differential DNA methylation and gene expression

Study/Year	Objective(s)	*Type of Study*	Evaluated Genes	Nominal Condition(s) of Interest/Periodontitis Definition Criteria (Clinical Parameters)	Conflict of Interest
Oliveira N.F.P. et al., 2009 [26]	To investigate the methylation status in the gene promoter of *IL8* in cells of the oral epithelium, gingival tissue and blood leucocytes of healthy subjects and to compare it with the findings in cells of smokers and non-smokers with periodontitis. We also analysed the levels of *IL8* mRNA in these groups.	Cross-sectional study	*IL8*	Periodontitis / At least three teeth exhibiting sites ⩾ 5 mm CAL, at least two different quadrants	There are no conflicts of interest associated with this work.
Ishida K et al., 2012 [28]	To assess the potential alteration in the methylation pattern of IL6 gene promoter in individuals with Rheumatoid arthritis, periodontitis, and healthy controls.	Cross-sectional study	*IL6*	Periodontitis or Rheumatoid Arthritis/ Sites with probing depth (PD) ⩾ 4 mm	Report no conflicts of interest related to this study.
Kojima A. et al., 2016 [27]	To evaluate potential alteration in the methylation pattern of the TNF a promoter in blood cells from Japanese adults with P and RA, by comparison with the age-, gender- and smoking status-matched healthy controls.	Cross-sectional study	*TNF* (promoter)	Periodontitis or Rheumatoid Arthritis // Sites with probing depth (PD) ⩾ 4 mm	Declare no conflict of interest
Shaddox L.M. et al., 2017 [25]	To examine the role of DNA methylation status in the promoter regions of genes involved in TLR signaling pathways in Periodontitis patients, potentially providing a link between methylation pattern and disease as well as the degree of inflammatory hyper-responsiveness.	Cross-sectional study	*CD14, FADD, HRAS, HSPA1A, HSPD1, IL6R, IRAK1, IRAK2, IRF1, IRF3, IRF8, MAP3K7, MYD88, PPARA, RIPK2, TBK1, TLR2, TLR5, TOLLIP, TRAF6, UBE2N, UBE2V1, EP_SEC, EP_DEC*<br><br>Selected after the screening: *FADD, MAP3K7, MYD88, PPARA, IRAK1, RIPK2,* and *IL6R*	Periodontitis/CAL ⩾ 4 mm local-ized at least two teeth (first molar)	Declare that they have no competing interests.
Kurushima Y, et al., 2019 [29]	To investigate epigenomic variation associated with periodontal disease using a twofold approach.	Cross-sectional study	Genome wide analysis<br><br>Locy focused analysis: *NIN, ABHD12B, WHAMM, KCNK1, DAB2IP, CLEC19A, TRA, TM9SF2P, GGTA2P, IFI16, RBMS3, C1QTNF7, TSNARE, HPVC1, SLC15A4, PKP2, SNRPN, IL8, CD44, CXCL1, IL6ST, CCR1, MMP7, MMP13, MMP3, TLR9, IL18, IFNB1, GLT6D1, IL1B, IL1RN, IL6, IL10, VDR,* CD14, TLR4, MMP1<br><br>RNA-sequencing	Self-reported periodontitis traits/<br>* "Have you ever had the condition of gum bleeding"<br>* "Have you ever had the condition of gum decay or loose teeth"	Declare that they have no competing interests.

			ZNF804A VDR, IL6ST, TMCO6, IL1RN, CD44, IL1B, WHAMM, and *CXCL1*		
Hernández H.G. et al., 2021 [17]	To identify the epigenome-wide DNA methylation patterns in peripheral leukocytes of patients with periodontitis in comparison with periodontally healthy controls, correcting for the effects of cell population distribution, primarily measured via direct cell counting, and complementing it with a recently available optimized estimation of cell counts.	Cross-sectional study	Genome wide analysis	Periodontitis/NR	The authors have no financial conflict with the subject matter

TLR, Toll- like receptors; NR, not reported; CAL, Clinical attachment loss.
