# Supplementary material for: Biomarkers of Periodontitis and Its Differential DNA Methylation and Gene Expression in Immune Cells: A Systematic Review
Source: Int J Mol Sci. 2022 Oct 10;23(19):12042. doi: 10.3390/ijms231912042 (PMC9570497; doi:10.3390/ijms231912042)
Supplement: Supplementary file 1 [file ijms-23-12042-s001.zip › Tabla S2.pdf]

Table S2. General characteristics Studies assessing Peripheral blood mononuclear cells (PBMCs) or subcomponents (lymphocytes and monocytes) differential gene expression

**Author/Year**	**Objective(s)**	***Type of Study***	**Evaluated Genes**	**Nominal Condition(s) of Interest / Periodontitis Definition Criteria**	Conflict of Interest
Sørensen L.K. et al., 2008 [32]	To identify **differentially expressed candidate genes** in PBMCs from small groups of untreated subjects with Periodontitis and then to validate the differentially expressed genes using reverse transcription-polymerase chain reaction (RT PCR) on PBMCs from another independent group of subjects with Periodontitis<br><br>To identify possible disease-specific gene **expression** characteristics of subjects with periodontitis relative to subjects with chronic inflammation in general.	Cross-sectional study	Genome wide analysis	Periodontitis/CAL ⩾ 4 mm local-ized at least two teeth or CAL ⩾ 4 mm at least three teeth.	Report no conflicts of interest
Gonzales J.R. et al., 2012 [33]	To analyze the **expression** and production of *IL2, IFNG, IL4* and *IL13* in CD4+ cells from the peripheral blood of patients with Periodontitis	cross-sectional study	Th1 and Th2 cytokines (*IL2, IFNG, IL4* and *IL13*)	Periodontitis/PPD and CAL ⩾ 5 mm on at least one interproximal site affecting at least three teeth other than the first molars and incisors.	Declare that there are no conflicts of interest
Liu Y.-Z. et al., 2016 [34]	To reveal new functional genes and pathways for Periodontitis at **monocyte transcriptomic** level.	cross-sectional study	Genome wide analysis	Periodontitis/ CAL ⩾ 5 mm	Declare that they have no competing interests
Corbi S.C.T. et al., 2020 [35]	To identify specific **gene expression** signatures from circulating lymphocytes and monocytes (in joint) to reveal potential biomarkers (compared to healthy patients)	Cross-sectional study	Genome wide analysis	Periodontitis alone or associated with dyslipidemia and diabetes mellitus type II /PD ⩾6 mm and CAL ⩾ 4 mm in at least 4 non-adjacent teeth.	Declare no competing interests.
Gonçalves Fernandes J et al., 2020 [36]	To investigate the **gene expression** of key TLR pathway genes and miRNA regulators in unstimulated PBMCs obtained from patients diagnosed with periodontitis as to identify potential candidate genes and gene regulators that may be altered in the pathogenesis of this disease	Cross-sectional study	84 genes from TLR pathway RT² Profiler PCR Arrays<br><br>84 genes miRNA gnes from miScript PCR Arrays Human Immunopathology	Periodontitis/At least 2 sites with CAL >2 mm and radiographic bone loss on first molar or incisor.	Declare no conflict of interest

PBMCs, peripheral blood mononuclear cells; TLR, Toll- like receptors; Th, T helper cells; PD, periodontal depth; CAL, clinical attachment loss; PPD, Probing pocket depths .
