# Supplementary material for: Biomarkers of Periodontitis and Its Differential DNA Methylation and Gene Expression in Immune Cells: A Systematic Review
Source: Int J Mol Sci. 2022 Oct 10;23(19):12042. doi: 10.3390/ijms231912042 (PMC9570497; doi:10.3390/ijms231912042)
Supplement: Supplementary file 1 [file ijms-23-12042-s001.zip › Tabla S3.pdf]

Table S3. Methodological and results description of studies assessing peripheral blood leucocytes cells (PBLCs) differential DNA methylation and gene expression

Authors	Subject/ Population	Comparison	Expression Technique Source	Methylation/Expression Technique	Main Results	Systemic Biomarkers meth / mRNA
Oliveira N.F.P. et al., 2009 [26]	13 smokers with periodontitis 13 non smokers with periodontitis	13 healthy (never smoked) control subjects (absence of CAL and no sites with probing depth>3 mm)	N.A	Methylation-specific PCR (MSP)	No significant differences among groups were observed in blood cells. Most individuals in the control group showed an *IL-8* methylation status positive to both conditions, methylated and unmethylated.	No DNA methylation/transcriptional expression biomarkers found for *IL8*
Ishida K et al., 2012 [28]	30 patients with RA and 30 patients with Periodontitis	30 age-, sex-, and smoking status–balanced healthy controls	N.A	Direct bisulfite sequencing (*IL6* promoter)	* The region of *IL6* gene promoter from -1200 to +27 bp was shown to contain 19 CpG motifs. The methylation levels of the CpG motif at -74 bp were significantly lower in patients with RA and P than those in controls (P = 0.0001).<br>* Both levels of serum *IL6* and *IL6* production by mononuclear cells were significantly different between individuals with and without the methylation at -74 bp (P = 0.03).<br>* The +19 bp motif exhibited differential levels of the methylation among the groups, which was not associated with serum levels of *IL6*. The other 17 CpG motifs exhibited comparable levels of the methylation between the groups.<br>*At -74 bp, all samples in the RA and P groups were unmethylated, whereas eight (26.7%) and 22 (73.3%) samples in the H group were partially methylated and unmethylated, respectively. At +19 bp, all samples in the RA and H groups were unmethylated, whereas seven (23.3%) and 23 (76.7%) samples in the P group were partially methylated and unmethylated, respectively.	Hypometh *IL6*

Kojima A. et al., 2016 [27]	30 patients with periodontitis(only)<br>30 patients with rheumatoid arthritis (RA) /<br>Japanese adults	30 race-matched healthy controls	N.A	Direct bisulfite sequencing (Signal correction using ESME)	* 12 CpGs dinucleotides were identified in *TNF* promoter fragment from -343 to +57 bp. The periodontitis group showed a significantly higher methylation rate and frequency at -72 bp than the healthy controls group.<br><br>* The levels of *TNF* produced were significantly different between individuals with and without methylation at -163 bp<br>* The RA group exhibited significantly higher methylation rates at seven CpG motifs (-302, -163, -119, -72, -49, -38 and +10 bp), and significantly higher methylation frequencies at six CpG motifs (-63, -119, -72, -49, -38 and +10 bp), than the healthy controls group.	Hypermeth *TNF*
Shaddox L.M. et al., 2017 [25]	20 periodontitis/<br>African American<br>5-25 years old<br>(10 initial and 10 advanced stages of the disease)	20 healthy unrelated controls	N.A	EpiTect Methyl II PCR<br><br>Array Human Toll-Like Receptor Signaling Pathway Signature Panel (Pyrosequencing)	* Subjects with early stages of periodontitis presented hypermethylation of both the upregulating (*MAP3K7, MYD88, IL6R, and RIPK2)* and downregulating (*FADD, IRAK1BP1*, and *PPARA*) genes while advances stages presented hypomethylation of these genes in comparison to early stages of periodontitis.<br>* Further analysis on CpG sites with significant differences in methylation status correlates with an increased pro-inflammatory cytokine profile for P patients.<br>* Positive correlations of *FADD* positions 2 and 5 and pro-inflammatory (*GM-CSF, INFG, IL1β, IL6, TNF*, among others).	Early stages of the disease<br>Hypermeth *MAP3K7*<br>Hypermeth *MYD88*<br>Hypermeth *IL6R*<br>Hypermeth *RIPK2* (early stages of the disease)<br>Hypermeth *IRAK1BP1*<br>Hypermeth *PPARA*<br>Hypermeth *FADD*<br>Advanced stages of the disease<br>Hypometh *RIPK2*<br>Hypomet *MAP3K7*(at positions 1 and 3)<br>Hypometh*MYD88* (at positions 1 and 5)

						Hypometh<br>*IRAK1BP1* (at positions 1 and 3)<br>Hypomet *PPARA* (at position 2)
Kurushima Y, et al., 2019 [29]	* Patientes wirh Selfreported periodontitis<br>- Positive gingival bleeding trait : 269 felmale subjects (from 528 female individuals)<br>- Positive for tooth movility trait: 121 female subjects(from 492 female individuals)**<br><br>* RNA-sequencing (384 subjects)<br>- Positive gingival bleeding trait: 342 (from 384 female individuals)<br>- Positive for tooth movility trait: 335 (from 384 female individuals)	*Subjects without self-reported periodontitis"<br>- Positive gingival bleeding trait : 259 felmale subjects (from 528 female individuals)<br>- Positive for tooth movility trait : 371 female subjects(from 492 female individuals)**<br><br>* RNA-sequencing (384 subjects)<br>- Negative gingival bleeding trait: 42 (from 384 female individuals)<br>- Negative for tooth movility trait: 49 (from 384 female individuals)	RNA-sequencing	The Infinium Human Methylation 450 BeadChip	* In blood, 26 CpG sites (tooth mobility) and 15 CpG sites (gingival bleeding) were nominally significantly associated with dental phenotypes.<br>* CpG sites that were nominally significantly associated with both periodontal traits mapped to eight genes of the candidates genes (*VDR, IL6ST, TMCO6, IL1RN, CD44, IL1B, WHAMM, and CXCL1*).<br><br>* One CpG site located on the 5′ UTR of *ZNF804A* hypo-methylated in participants who experienced gingival bleeding (cg21245277; beta = − 0.33, p value = 7.17e−8, FDR = 0.03.<br><br>* RNA-sequencing gene expression data in 342 twins identified two exons in *ZNF804A*, which are expressed in blood. One exon showed a nominally. significant association with the presence of gingival bleeding (beta = 2.17e−6, p value = 0.018) in a model adjusting for age, smoking status, family structure, and technical covariates.<br><br>* Subjects who experienced gingival bleeding had lower levels of DNA methylation in the 5′ UTR of this gene and higher levels of exon expression compared to controls, suggesting negative epigenetic regulatory effects of cg21245277 on exon expression (correlation = − 0.15, p value = 0.11).<br><br>* The two most associated CpG sites were located in the gene body of the *IQCE* gene (cg08157914; beta = 0.38, p value = 6.85e −8, FDR < 0.001 and in the gene body of	DNA Methylation in blood<br><br>Hypometh *ZNF804A* in gingival bleeding.<br>Hypermeth *IQCE* in tooth mobility<br>Hypometh *XKR6* in tooth mobility<br><br>*VDR, IL6ST, TMCO6, IL1RN, CD44, IL1B, WHAMM, and CXCL1*[£]<br><br>mRNA Expression in Blood<br>↑ mRNA *ZNF804A* in gingival bleeding<br><br>*WHAMM, TMCO6*[£]<br><br>[£] the direction of methylation

					the *XKR6* gene (cg11051055; beta = − 0.49, p value = 1.53e−8, FDR = 0.003).<br><br>* Three (cg18353661, cg23681110, cg08453750) out of 58 CpG sites were located in or near to the genes that also showed exon expression levels with a nominally significant association with tooth.	diiference was not reported
Hernández H.G. et al., 2021 [17]	8 periodontitis patients	8 periodontally healthy subjects	N.A	Illumina MethylationEPIC BeadChip (IMEB)	* 81 differentially hypermethylated genes<br>* 21 differentially hypomethylated genes<br>* The intersection analysis showed *ZNF718* and *HOXA4* were differentially hypermethylated and *ZFP57* was differentially hypomethylated in periodontitis.<br>* The functional enrichment analysis found clearly immune response related ontologies such as "detection of bacterium" and "antigen processing and presentation".<br>* Regarding the differential hypermethylation found in the DMP analysis, the top differentially hypermethylated position corresponds to *ZNF718*.	Hypermeth *ZNF718*<br>Hypermeth *HOXA4*<br>Hypometh *ZFP57*

Meth, methylation; ESME, Sanger/epigenetic sequencing methylation analysis; CAL, clinical attachement loss; Hypometh, Differentially Hypomethylated; Hypermeth, Differentially Hypermethylated; Red arrow ↑ and green arrow ↓denote increase or decrease in mRNA expression; Rheumatoid arthritis, AR; Periodontitis, P; H, healthy; DMP, differentially methylated position; bp, base pair; CpG, Cytosine-phosphate-guanine; † The direction of methylation difference was not reported. ‡ Correspondence of DNA methylation with gene expression for ZNF804A.
