# Supplementary material for: Biomarkers of Periodontitis and Its Differential DNA Methylation and Gene Expression in Immune Cells: A Systematic Review
Source: Int J Mol Sci. 2022 Oct 10;23(19):12042. doi: 10.3390/ijms231912042 (PMC9570497; doi:10.3390/ijms231912042)
Supplement: Supplementary file 1 [file ijms-23-12042-s001.zip › Tabla S4.pdf]

**Tabla S4.** Methodological and results description of studies assessing polymorphonuclears (PMNs) cells or subcomponents (neutrophils) differential gene expression

| Authors | Subject/ Population | Comparison | Cell Type(s)/ Source | Methylation/ Expression Technique | Main Results | Systemic Biomarkers meth/mRNA |
|---|---|---|---|---|---|---|
| Wright HJ et al., 2008 [30] | 19 Patientes with periodontitis (19, 36- 61 years) (baseline and 3 months after periodontal treatment*) | 19 Age- and gender-matched periodontally healthy control subjects ( 37– 62 years) | Neutrophils (discontinuous Percoll gradient isolation)/ peripheral blood | HG_U133A microarrays (Affymetrix)<br><br>semi-quantitative RT-PCR | Pairwise analysis of hybri dization data indicated that of the 5680 genes detected as being present in both targets, 163 genes (2.87% of detected genes) were 2-fold or greater differentially expressed between healthy and periodontitis patient samples.<br><br>14 were more highly expressed in neutrophils from healthy patients than those with periodontitis (age- and gender-matched)<br><br>149 were upregulated in periodontitis patient neutrophils relative to healthy patients.<br><br>RT-PCR analysis<br>The gene expression data confirmed that transcript levels for all six genes (MX1, IFIT4, G1P2, IFIT1, CIG5, and IFI44-like) were increased. | ↑ mRNA *MX1, IFIT4, G1P2, IFIT1, CIG5,* and *IFI44-like* |
| Iwata T et al., 2009 [31] | 36 patients with periodontitis (age range: 16 to 41 years) | 36 systemically healthy control subjects (n = 36; age range: 21 to 39 years) | PMNs discontinuous gradient | RT-qPCR | Compared to healthy PMNs, Periodontitis PMNs expressed significantly more CP mRNA. | ↑ mRNA *CP* |

HG, Human genome; CP, ceruloplasmin. Green arrow ↑denote increase in mRNA expression.
