# Supplementary material for: Biomarkers of Periodontitis and Its Differential DNA Methylation and Gene Expression in Immune Cells: A Systematic Review
Source: Int J Mol Sci. 2022 Oct 10;23(19):12042. doi: 10.3390/ijms231912042 (PMC9570497; doi:10.3390/ijms231912042)
Supplement: Supplementary file 1 [file ijms-23-12042-s001.zip › Tabla S5.pdf]

Tabla S5. Methodological and results description of studies assessing Peripheral blood mononuclear cells (PBMCs) or subcomponents (lymphocytes and monocytes) differential gene expression

Authors	Subject/ Population	Comparison	Cell Type(s)/ Source	Methylation/ Expression Technique	Main Results	Systemic Biomarkers meth / mRNA
Sørensen L.K. et al., 2008 [32]	For microarray<br>5 Subjects with periodontally untreated:<br>For RT-PCR<br>45 subjects with periodontitis	For microarray<br>2 controls with healthy periodontium (no interproximal attachment loss and no clinical signs of oral inflammatory conditions)<br><br>For RT-PCR<br>25 healthy control subjects	Mononuclear cells(density centrifugation)/ peripheral blood	HG-U133A expression array<br><br>RT-PCR, qPCR	*The microarray analysis of genes in subjects with Periodontitis compared to healthy controls showed that 53 transcripts were expressed differentially.<br>* 52 transcripts increased.<br>* 1 transcript (interleukin-32 [*IL32*]) decreased.<br>* Toll like receptor (TLR) 2 gene was upregulated in sub jects with P compared to control.<br>* Gene expression was not affected significantly by differences in the numbers of monocytes (P = 0.71) or lymphocytes (P = 0.62).<br>* The relative expression of *MYOM2* and *TLR2* were statistically significant in P (P = 0.032 and P = 0.003, respectively) and confirmed by RT-PCR.<br>* *IL6*, *THBS1*, *IL1B*, *DEFA1* no reached statistically significant differences.<br>* Immune responses, response to external stimulus, apoptosis, cytokine activity, and chemotaxis were overrepresented significantly<br>* Fourteen genes were associated with immune responses	Periodontitis<br>↑mRNA *MYOM2*<br>↑ mRNA *TLR2*
Gonzales J.R. et al., 2012	20 Periodontitis	20 Non-periodontitis control subjects	CD4+ cells /	Real-time polymerase	* In the inactivated CD4+ cells:	In the inactivated CD4+ cells in periodontitis:

[33]			peripheral blood	chain reaction (RT PCR) (TaqMan®)	* In unstimulated CD4+ cells, there was a difference in *IL4* expression between P patients (17.8±3.6) vs. controls (41.5±2.1); being singnifally lower in P ($p<0.05$, Mann–Whitney U test).<br>* Expression of *IL-2* (10.5±2.6 RFU) and *IFNG* (5.7±1.8 RFU) was lower than expression of *IL4* in the cells of the controls.<br>* In the group of patients, the expression of *IL4* (17.8±3.6 RFU), *IL13* (19.1±2.8 RFU) and *IL2* (18.6±2.8 RFU) was higher than the expression of *IFNG* (4.9±0.2 RFU).<br>* In the same way, *IL2* expression in RFU (Relative Fluorescent Unities) was lower than expression of *IL4* in the cells from patients (10.5±2.6 RFU vs. *IFNG* 5.7±1.8 RFU).<br>* In activated CD4+ cells from controls *IFNG* and *IL2* expressions were increased	↓ mRNA *IL4*
Liu Y.-Z. et al., 2016 [34]	5 periodontitis subjects (non-smoking)	5 periodontally healthy	Mononuclear cells / peripheral blood	RNA-seq (Illumina TruSeq)<br><br>microarray dataset GSE6751	* At the whole gene (or whole non-coding RNA) level, we identified a total of 380 DE transcripts in P vs. control individuals, which achieved a PPEE (posterior probability of equal expression) of < 0.05 and a PPDE (posterior probability of differential expression) of > 0.95. Among these transcripts, 228 were upregulated and 152 were down-regulated in P vs. control individuals.	Periodontitis pathogenesis<br>↑*mRNA FACR, CLCN5 CUX1, RNASE3, REL, VNN2, SGMS2, GGT1, HLA-DOA, ME1*<br>↓ mRNA *PXN-AS1, URGCP, RPS20, FAM98A, XBP1, G3BP1, NFAT5* and *ZNF207.*<br><br>Endocytosis<br>↑mRNA *DENND1A, RUFY1, CORO1C, ASGR2, APP, DAB2, PICALM, CD36, AP1S2, CLEC7A, THBS1, CLCN5* and *RIN3*<br><br>Cytokine production<br>↑mRNA *NLRC4, G6PD, MYD88, TLR4, NLRP3* and *PTAFR*<br><br>Apoptosis

					* By comparing with the microarray dataset, we identified several P-associated novel genes (e.g., *FACR* and *CUX1*) that have functions to interact with invading microorganisms or enhance TNF production on lipopolysaccharide stimulation. DAVID analysis of both the RNA-seq and the microarray datasets leads to converging evidence supporting “endocytosis”, “cytokine production” and “apoptosis” as significant biological processes in P.	↑mRNA *ARHGEF2, SGK1, DNM1L, XIAP, UBE4B, CIDEB, STK17B, TRIO, NLRP3, BCL2L13, NCSTN, TNFRSF1A, PEA15, NLRC4, APP, GSN, HIPK3, BNIP3L, NLRP12,* and *THBS1*
					* Through differential expression analyses at both whole gene (or whole non-coding RNA) and isoform levels, we identified 380 DEx (diferencially expressed) transcripts and 5955 DEx isoforms with a PPEE (posterior probability of equal expression) of b0.05. Pervasive up-regulation of transcripts at isoform level in P vs. control individuals was observed, suggesting a more functionally active monocyte transcriptome for P.	
Corbi S.C.T. et al., 2020 [35]	24 periodontitis patients for Microarray expression (U133 Plus 2.0, Affimetrix) :<br>- 5 poorly controlled T2DM + dyslipidemia + periodontitis (T2DMpoorly-DL-P)<br>- 7 well-controlled T2DM with dyslipidemia and periodontitis (T2DMwell-DL-P)<br>- 6 well-controlled T2DM + dyslipidemia + periodontitis (DL-P)	For Microarray U133 Plus 2.0:<br>-6 systemically healthy individuals without periodontitis (H)<br>(homogeneity regarding biochemical, lipid and clinical periodontal parameters)<br><br>For RT-qPCR validation:<br>- 30 H	blood mono-nuclear cells (Lymphocytes and monocytes) / peripheral blood	Expression Microarray U133 Plus 2.0<br><br>RT-qPCR (validation)	* T2DMpoorly-DL-P versus H comparison, we identified 1374 up- and down-regulated DEG<br>* 869 DEG in the T2DMwell-DL-P versus H, 521 up- and down-regulated DEGs (DL-P versus H) and	* P<br>↓ mRNA *IGHG3*<br>↑ mRNA *ITGB2* and *HLADRB4.*<br><br>* T2DMpoorly+DL+P<br>↑ mRNA *TGFB1I1, VNN1*<br>↓ mRNA *HLADRB4* and *CXCL8*<br><br>* T2DMwell+DL+P<br>↓ mRNA *BPTF, PDE3B*<br>↑ mRNA *FN1*

	- 6 normoglycemic individuals + dyslipidemia + periodontitis (P)<br><br>120 periodontitis patients for RT-qPCR validation of selected DEGs:<br>- 30 T2DMpoorly-DL-P<br>- 30 T2DMwell-DL-P<br>- 30 DL-P<br>- 30 P				* 564 up- and down-regulated DEGs (H versus P).<br><br>RT-qPCR Validated DEGs<br>* T2DMpoorly-DL-P versus H:<br>Up regulated in patients: *TGFB1I1*, *VNN1*<br>Down regulated in patients: *HLADRB4* and *CXCL8*<br><br>* T2DMwell-DL-P versus H:<br>Down regulated in patients: *BPTF*, *PDE3B*<br>Up regulated in patients: *FN1*<br><br>* DL-P versus H:<br>Up regulated In patients: *DAB2*,<br>Down regulated in patients: *CD47* and *HLADRB4*<br>* P versus H:<br>Up regulated in patients: *IGHG3*<br>Down regulated in patients: *ITGB2* and *HLADRB4*.	* DL+P<br>↑ mRNA *DAB2*<br>↓ mRNA *CD47* and *HLADRB4*
Gonçalves Fernandes J et al., 2020 [36]	For array screning.<br>10 subjects with periodontitis for mRNA screening RT2 Profiler PCR Arrays (TLR pathway)<br>microRNA ARRAY<br>* 11 subjects with periodontitis for microRNA screening by miScript Immunopathology PCR arrays (Qiagen)<br><br>For gene expression validation:<br>* 29 periodontally healthy subjects for mRNA(qPCR)<br>* 31 periodontally healthy subjects for microRNAs(Qpcr)<br><br>African American subjects	For array screning.<br>* 9 control subjects for mRNA screening RT^2 Profiler PCR Arrays (TLR pathway)<br>* 11 control subject for microRNA screening by miScript Immunopathology PCR arrays (Qiagen)<br><br>For gene expression validation:<br>29 periodontally healthy subjects for mRNA(qPCR)<br>31 periodontally healthy subjects for microRNAs(Qpcr)	mononuclear cells (SepMate ™ Isolation method)/ peripheral blood	-RT^2 Profiler PCR Arrays (TLR pathway)<br><br>- miScript PCR Arrays Human Immunopathology<br><br>- RT-qPCR (validation)	* Five genes were significantly upregulated in Periodontitis patients compared to HC: *TLR2*, *TICAM-1 (TRIF)*, *IRAK1*, *FOS* and *CCL2*.<br><br>**miRNAs MIR9-1*, *MIR155*, *MIR203A*, *MIR147A*, *MIR182*, *MIR183*were significantly up-regulated in Periodontitis compared to HC.<br><br>*TLR2*	↑ mRNA *TLR2*, *TICAM-1 (TRIF)*, *IRAK1*, *FOS*, *CCL2*<br><br>↑ mRNA *miRNAs MIR9-1*, *MIR155*, *MIR203A*, *MIR147A*, *MIR182*, *MIR183*

HG, Human genome; Red arrow ↓ and green arrow ↑ denote increase or decrease in mRNA expression; Healthy control, HC; DAVID, Database for Annotation, Visualization and Integrated Discovery; RFU, relative fluorescence units.
