# Supplementary material for: Biomarkers of Periodontitis and Its Differential DNA Methylation and Gene Expression in Immune Cells: A Systematic Review
Source: Int J Mol Sci. 2022 Oct 10;23(19):12042. doi: 10.3390/ijms231912042 (PMC9570497; doi:10.3390/ijms231912042)
Supplement: Supplementary file 1 [file ijms-23-12042-s001.zip › Tabla S6.pdf]

Tabla S6. Candidate genes to be related to periodontitis identified in peripheral blood leukocytes from included studies

	Official symbol	*Official name*	*Funtion*
BPTF	BPTF	Bromodomain PHD Finger Transcription Factor	"Chromatin and facilitate access to DNA during DNA-templated processes such as DNA replication, transcription, and repair (PubMed:14609955, PubMed:28801535). The NURF-1 ISWI chromatin remodeling complex has a lower ATP hydrolysis rate than the NURF-5 ISWI chromatin remodeling complex (PubMed:28801535). Within the NURF-1 ISWI chromatin-remodeling complex, binds to the promoters of En1 and En2 to positively regulate their expression and promote brain development (PubMed:14609955). Histone-binding protein which binds to H3 tails trimethylated on 'Lys-4' (H3K4me3), which mark transcription start sites of active genes (PubMed:16728976, PubMed:16728978). Binds to histone H3 tails dimethylated on 'Lys-4' (H3K4Me2) to a lesser extent (PubMed:16728976, PubMed:16728978, PubMed:18042461). May also regulate transcription through direct binding to DNA or transcription factors (PubMed:10575013).
CCL2	CCL2	C-C Motif Chemokine Ligand 2	Acts as a ligand for C-C chemokine receptor CCR2 (PubMed:9837883, 10587439, 10529171). Signals through binding and activation of CCR2 and induces a strong chemotactic response and mobilization of intracellular calcium ions (PubMed:9837883, 10587439). Exhibits a chemotactic activity for monocytes and basophils but not neutrophils or eosinophils (PubMed:8627182, 9792674, 8195247). May be involved in the recruitment of monocytes into the arterial wall during the disease process of atherosclerosis (PubMed:8107690).
CIG5	RSAD2	Radical S-Adenosyl Methionine Domain Containing 2	interferon-inducible antiviral protein which plays a major role in the cell antiviral state induced by type I and type II interferon

			(PubMed:31812350). Catalyzes the conversion of cytidine triphosphate (CTP) to 3'-deoxy-3',4'-didehydro-CTP (ddhCTP) via a SAM-dependent radical mechanism (PubMed:29925952, 30872404). In turn, ddhCTP acts as a chain terminator for the RNA-dependent RNA polymerases from multiple viruses and directly inhibits viral replication (PubMed:29925952). Therefore, inhibits a wide range of DNA and RNA viruses, including human cytomegalovirus (HCMV), hepatitis C virus (HCV), west Nile virus (WNV), dengue virus, sindbis virus, influenza A virus, sendai virus, vesicular stomatitis virus (VSV), zika virus, and human immunodeficiency virus (HIV-1) (PubMed:29925952, 30587778, 31921110, 30934824). Promotes also TLR7 and TLR9-dependent production of IFN-beta production in plasmacytoid dendritic cells (pDCs) by facilitating 'Lys-63'-linked ubiquitination of IRAK1 by TRAF6 (PubMed:30872404). Plays a role in CD4+ T-cells activation and differentiation. Facilitates T-cell receptor (TCR)-mediated GATA3 activation and optimal T-helper 2 (Th2) cytokine production by modulating NFKB1 and JUNB activities. Can inhibit secretion of soluble proteins.
CUX1	CUX1	Cut Like Homeobox 1	May be involved in intra-Golgi retrograde transport. CASP_HUMAN,Q13948. Transcription factor involved in the control of neuronal differentiation in the brain. Regulates dendrite development and branching, and dendritic spine formation in cortical layers II-III. Also involved in the control of synaptogenesis. In addition, it has probably a broad role in mammalian development as a repressor of developmentally regulated gene expression. May act by preventing binding of positively-activing CCAAT factors to promoters. Component of nf-munr repressor; binds to the matrix attachment regions (MARs) (5' and 3') of the immunoglobulin heavy chain enhancer. Represses T-cell receptor (TCR) beta enhancer function by binding to MARbeta, an ATC-rich DNA sequence located upstream of the TCR beta enhancer. Binds to the TH enhancer; may require the basic helix-loop-helix protein TCF4 as a coactivator.

			CUX1_HUMAN,P39880. [CDP/Cux p110]: Plays a role in cell cycle progression, in particular at the G1/S transition. As cells progress into S phase, a fraction of CUX1 molecules is proteolytically processed into N-terminally truncated proteins of 110 kDa. While CUX1 only transiently binds to DNA and carries the CCAAT-displacement activity, CDP/Cux p110 makes a stable interaction with DNA and stimulates expression of genes such as POLA1.
CXCL8	CXCL8	C-X-C Motif Chemokine Ligand 8	Chemotactic factor that mediates inflammatory response by attracting neutrophils, basophils, and T-cells to clear pathogens and protect the host from infection (PubMed:7636208, 18692776). Also plays an important role in neutrophil activation (PubMed:9623510, 2145175). Released in response to an inflammatory stimulus, exerts its effect by binding to the G-protein-coupled receptors CXCR1 and CXCR2, primarily found in neutrophils, monocytes and endothelial cells (PubMed:1840701, 1891716). G-protein heterotrimer (alpha, beta, gamma subunits) constitutively binds to CXCR1/CXCR2 receptor and activation by IL8 leads to beta and gamma subunits release from Galpha (GNAI2 in neutrophils) and activation of several downstream signaling pathways including PI3K and MAPK pathways (PubMed:8662698, 11971003).
DAB2	DAB2	DAB Adaptor Protein 2	Adapter protein that functions as clathrin-associated sorting protein (CLASP) required for clathrin-mediated endocytosis of selected cargo proteins. Can bind and assemble clathrin, and binds simultaneously to phosphatidylinositol 4,5-bisphosphate (PtdIns(4,5)P2) and cargos containing non-phosphorylated NPXY internalization motifs, such as the LDL receptor, to recruit them to clathrin-coated pits. Can function in clathrin-mediated endocytosis independently of the AP-2 complex. Involved in endocytosis of integrin beta-1; this function seems to redundant with the AP-2 complex and seems to require DAB2 binding to endocytosis accessory EH domain-containing proteins such as EPS15, EPS15L1 and ITSN1. Involved in endocytosis of cystic fibrosis transmembrane conductance regulator/CFTR. Involved in

			endocytosis of megalin/LRP2 lipoprotein receptor during embryonal development. Required for recycling of the TGF-beta receptor.<br>Involved in CFTR trafficking to the late endosome.<br>Involved in several receptor-mediated signaling pathways.<br>Involved in TGF-beta receptor signaling and facilitates phosphorylation of the signal transducer SMAD2. Mediates TFG-beta-stimulated JNK activation. May inhibit the canoniocal Wnt/beta-catenin signaling pathway by stabilizing the beta-catenin destruction complex through a competing association with axin preventing its dephosphorylation through protein phosphatase 1 (PP1). Sequesters LRP6 towards clathrin-mediated endocytosis, leading to inhibition of Wnt/beta-catenin signaling. May activate non-canonical Wnt signaling.<br>In cell surface growth factor/Ras signaling pathways proposed to inhibit ERK activation by interrupting the binding of GRB2 to SOS1 and to inhibit SRC by preventing its activating phosphorylation at 'Tyr-419'. Proposed to be involved in modulation of androgen receptor (AR) signaling mediated by SRC activation; seems to compete with AR for interaction with SRC. Plays a role in the CSF-1 signal transduction pathway. Plays a role in cellular differentiation.<br>Involved in cell positioning and formation of visceral endoderm (VE) during embryogenesis and proposed to be required in the VE to respond to Nodal signaling coming from the epiblast. Required for the epithelial to mesenchymal transition, a process necessary for proper embryonic development. May be involved in myeloid cell differentiation and can induce macrophage adhesion and spreading. May act as a tumor suppressor.
FCAR	FCAR	Fc Alpha Receptor	Binds to the Fc region of immunoglobulins alpha.<br>Mediates several functions including cytokine production.
FN1	FN1	Fibronectin 1	Fibronectins bind cell surfaces and various compounds including collagen, fibrin, heparin, DNA, and actin (PubMed:3024962, 3900070, 3593230, 7989369). Fibronectins are involved in cell adhesion, cell

			motility, opsonization, wound healing, and maintenance of cell shape (PubMed:3024962, 3900070, 3593230, 7989369). Involved in osteoblast compaction through the fibronectin fibrillogenesis cell-mediated matrix assembly process, essential for osteoblast mineralization (By similarity). Participates in the regulation of type I collagen deposition by osteoblasts (By similarity). Acts as a ligand for the LILRB4 receptor, inhibiting FCGR1A/CD64-mediated monocyte activation (PubMed:34089617).<br>[Anastellin]: Binds fibronectin and induces fibril formation. This fibronectin polymer, named superfibronectin, exhibits enhanced adhesive properties. Both anastellin and superfibronectin inhibit tumor growth, angiogenesis and metastasis. Anastellin activates p38 MAPK and inhibits lysophospholipid signaling.
FOS	FOS	Fos Proto-Oncogene, AP-1 Transcription Factor Subunit	Nuclear phosphoprotein which forms a tight but non-covalently linked complex with the JUN/AP-1 transcription factor. In the heterodimer, FOS and JUN/AP-1 basic regions each seems to interact with symmetrical DNA half sites. On TGF-beta activation, forms a multimeric SMAD3/SMAD4/JUN/FOS complex at the AP1/SMAD-binding site to regulate TGF-beta-mediated signaling. Has a critical function in regulating the development of cells destined to form and maintain the skeleton. It is thought to have an important role in signal transduction, cell proliferation and differentiation. In growing cells, activates phospholipid synthesis, possibly by activating CDS1 and PI4K2A. This activity requires Tyr-dephosphorylation and association with the endoplasmic reticulum.
G1P2	ISG15	ISG15 Ubiquitin Like Modifier	Ubiquitin-like protein which plays a key role in the innate immune response to viral infection either via its conjugation to a target protein (ISGylation) or via its action as a free or unconjugated protein. ISGylation involves a cascade of enzymatic reactions involving E1, E2, and E3 enzymes which catalyze the conjugation of ISG15 to a lysine residue in the target protein (PubMed:33727702). Its target proteins include IFIT1, MX1/MxA, PPM1B, UBE2L6, UBA7, CHMP5,

			CHMP2A, CHMP4B and CHMP6. Isgylation of the viral sensor IFIH1/MDA5 promotes IFIH1/MDA5 oligomerization and triggers activation of innate immunity against a range of viruses, including coronaviruses, flaviviruses and picornaviruses (PubMed:33727702). Can also isgylate: EIF2AK2/PKR which results in its activation, DDX58/RIG-I which inhibits its function in antiviral signaling response, EIF4E2 which enhances its cap structure-binding activity and translation-inhibition activity, UBE2N and UBE2E1 which negatively regulates their activity, IRF3 which inhibits its ubiquitination and degradation and FLNB which prevents its ability to interact with the upstream activators of the JNK cascade thereby inhibiting IFNA-induced JNK signaling. Exhibits antiviral activity towards both DNA and RNA viruses, including influenza A, HIV-1 and Ebola virus. Restricts HIV-1 and ebola virus via disruption of viral budding. Inhibits the ubiquitination of HIV-1 Gag and host TSG101 and disrupts their interaction, thereby preventing assembly and release of virions from infected cells. Inhibits Ebola virus budding mediated by the VP40 protein by disrupting ubiquitin ligase activity of NEDD4 and its ability to ubiquitinate VP40. ISGylates influenza A virus NS1 protein which causes a loss of function of the protein and the inhibition of virus replication. The secreted form of ISG15 can: induce natural killer cell proliferation, act as a chemotactic factor for neutrophils and act as a IFN-gamma-inducing cytokine playing an essential role in antimycobacterial immunity. The secreted form acts through the integrin ITGAL/ITGB2 receptor to initiate activation of SRC family tyrosine kinases including LYN, HCK and FGR which leads to secretion of IFNG and IL10; the interaction is mediated by ITGAL (PubMed:29100055).
HLADRB4	HLADRB4	Major Histocompatibility Complex, Class II, DR Beta 4	Binds peptides derived from antigens that access the endocytic route of antigen presenting cells (APC) and presents them on the cell surface for recognition by the CD4 T-cells. The peptide binding cleft accommodates peptides of 10-30 residues. The peptides presented by

MHC class II molecules are generated mostly by degradation of proteins that access the endocytic route, where they are processed by lysosomal proteases and other hydrolases. Exogenous antigens that have been endocytosed by the APC are thus readily available for presentation via MHC II molecules, and for this reason this antigen presentation pathway is usually referred to as exogenous. As membrane proteins on their way to degradation in lysosomes as part of their normal turn-over are also contained in the endosomal/lysosomal compartments, exogenous antigens must compete with those derived from endogenous components. Autophagy is also a source of endogenous peptides, autophagosomes constitutively fuse with MHC class II loading compartments. In addition to APCs, other cells of the gastrointestinal tract, such as epithelial cells, express MHC class II molecules and CD74 and act as APCs, which is an unusual trait of the GI tract. To produce a MHC class II molecule that presents an antigen, three MHC class II molecules (heterodimers of an alpha and a beta chain) associate with a CD74 trimer in the ER to form a heterononamer. Soon after the entry of this complex into the endosomal/lysosomal system where antigen processing occurs, CD74 undergoes a sequential degradation by various proteases, including CTSS and CTSL, leaving a small fragment termed CLIP (class-II-associated invariant chain peptide).The removal of CLIP is facilitated by HLA-DM via direct binding to the alpha-beta-CLIP complex so that CLIP is released. HLA-DM stabilizes MHC class II molecules until primary high affinity antigenic peptides are bound. The MHC II molecule bound to a peptide is then transported to the cell membrane surface. In B-cells, the interaction between HLA-DM and MHC class II molecules is regulated by HLA-DO. Primary dendritic cells (DCs) also to express HLA-DO. Lysosomal microenvironment has been implicated in the regulation of antigen loading into MHC II molecules, increased

			acidification produces increased proteolysis and efficient peptide loading.
HOXA4	HOXA4	Homeobox A4	Sequence-specific transcription factor which is part of a developmental regulatory system that provides cells with specific positional identities on the anterior-posterior axis.<br>Binds to sites in the 5'-flanking sequence of its coding region with various affinities. The consensus sequences of the high and low affinity binding sites are 5'-TAATGA[CG]-3' and 5'-CTAATTTT-3'.
IFI44	IFI44	Interferon Induced Protein 44	This protein aggregates to form microtubular structures.
IFIT1	IFIT1	Interferon Induced Protein With Tetratricopeptide Repeats 1	Interferon-induced antiviral RNA-binding protein that specifically binds single-stranded RNA bearing a 5'-triphosphate group (PPP-RNA), thereby acting as a sensor of viral single-stranded RNAs and inhibiting expression of viral messenger RNAs. Single-stranded PPP-RNAs, which lack 2'-O-methylation of the 5' cap and bear a 5' triphosphate group instead, are specific from viruses, providing a molecular signature to distinguish between self and non-self mRNAs by the host during viral infection. Directly binds PPP-RNA in a non-sequence-specific manner. Viruses evolved several ways to evade this restriction system such as encoding their own 2'-O-methylase for their mRNAs or by stealing host cap containing the 2'-O-methylation (cap snatching mechanism). Exhibits antiviral activity against several viruses including human papilloma and hepatitis C viruses.
IFIT4	IFIT3	Interferon Induced Protein With Tetratricopeptide Repeats 3	IFN-induced antiviral protein which acts as an inhibitor of cellular as well as viral processes, cell migration, proliferation, signaling, and viral replication. Enhances MAVS-mediated host antiviral responses by serving as an adapter bridging TBK1 to MAVS which leads to the activation of TBK1 and phosphorylation of IRF3 and phosphorylated IRF3 translocates into nucleus to promote antiviral gene transcription. Exhibits an antiproliferative activity via the up-regulation of cell cycle negative regulators CDKN1A/p21 and CDKN1B/p27. Normally, CDKN1B/p27 turnover is regulated by COPS5, which binds

			CDKN1B/p27 in the nucleus and exports it to the cytoplasm for ubiquitin-dependent degradation. IFIT3 sequesters COPS5 in the cytoplasm, thereby increasing nuclear CDKN1B/p27 protein levels. Up-regulates CDKN1A/p21 by down-regulating MYC, a repressor of CDKN1A/p21. Can negatively regulate the apoptotic effects of IFIT2.
IFN	IFNG	Interferon Gamma	Type II interferon produced by immune cells such as T-cells and NK cells that plays crucial roles in antimicrobial, antiviral, and antitumor responses by activating effector immune cells and enhancing antigen presentation (PubMed:16914093, 8666937). Primarily signals through the JAK-STAT pathway after interaction with its receptor IFNGR1 to affect gene regulation (PubMed:8349687). Upon IFNG binding, IFNGR1 intracellular domain opens out to allow association of downstream signaling components JAK2, JAK1 and STAT1, leading to STAT1 activation, nuclear translocation and transcription of IFNG-regulated genes. Many of the induced genes are transcription factors such as IRF1 that are able to further drive regulation of a next wave of transcription (PubMed:16914093). Plays a role in class I antigen presentation pathway by inducing a replacement of catalytic proteasome subunits with immunoproteasome subunits (PubMed:8666937). In turn, increases the quantity, quality, and repertoire of peptides for class I MHC loading (PubMed:8163024). Increases the efficiency of peptide generation also by inducing the expression of activator PA28 that associates with the proteasome and alters its proteolytic cleavage preference (PubMed:11112687). Up regulates as well MHC II complexes on the cell surface by promoting expression of several key molecules such as cathepsins B/CTSB, H/CTSH, and L/CTSL (PubMed:7729559). Participates in the regulation of hematopoietic stem cells during development and under homeostatic conditions by affecting their development, quiescence, and differentiation (By similarity).
IGHG3	IGHG3	Immunoglobulin Heavy Constant Gamma 3 (G3m Marker)	Constant region of immunoglobulin heavy chains. Immunoglobulins, also known as antibodies, are membrane-bound or secreted

			glycoproteins produced by B lymphocytes. In the recognition phase of humoral immunity, the membrane-bound immunoglobulins serve as receptors which, upon binding of a specific antigen, trigger the clonal expansion and differentiation of B lymphocytes into immunoglobulins-secreting plasma cells. Secreted immunoglobulins mediate the effector phase of humoral immunity, which results in the elimination of bound antigens (PubMed:22158414, 20176268). The antigen binding site is formed by the variable domain of one heavy chain, together with that of its associated light chain. Thus, each immunoglobulin has two antigen binding sites with remarkable affinity for a particular antigen. The variable domains are assembled by a process called V-(D)-J rearrangement and can then be subjected to somatic hypermutations which, after exposure to antigen and selection, allow affinity maturation for a particular antigen. (PubMed:17576170, 20176268).
IL-13	IL13	Interleukin 13	Cytokine that plays important roles in allergic inflammation and immune response to parasite infection (PubMed:8096327, 8097324). Synergizes with IL2 in regulating interferon-gamma synthesis (PubMed:8096327). Stimulates B-cell proliferation, and activation of eosinophils, basophils, and mast cells (PubMed:7903680, 8759755). Plays an important role in controlling IL33 activity by modulating the production of transmembrane and soluble forms of interleukin-1 receptor-like 1/IL1RL1 (By similarity). Displays the capacity to antagonize Th1-driven proinflammatory immune response and downregulates synthesis of many proinflammatory cytokines including IL1, IL6, IL10, IL12 and TNF-alpha through a mechanism that partially involves suppression of NF-kappa-B (By similarity). Functions also on nonhematopoietic cells, including endothelial cells where it induces vascular cell adhesion protein 1/VCAM1, which is important in the recruitment of eosinophils (PubMed:8639787). Exerts its biological effects through its receptors which comprises the IL4R chain and the IL13RA1 chain, to activate JAK1 and TYK2,

			leading to the activation of STAT6 (PubMed:9013879). Aside from IL13RA1, another receptor IL13RA2 acts as a high affinity decoy for IL13 and mediates internalization and depletion of extracellular IL13 (PubMed:21622864).
IL-2	IL2	Interleukin 2	Cytokine produced by activated CD4-positive helper T-cells and to a lesser extend activated CD8-positive T-cells and natural killer (NK) cells that plays pivotal roles in the immune response and tolerance (PubMed:6438535). Binds to a receptor complex composed of either the high-affinity trimeric IL-2R (IL2RA/CD25, IL2RB/CD122 and IL2RG/CD132) or the low-affinity dimeric IL-2R (IL2RB and IL2RG) (PubMed:16293754, 16477002). Interaction with the receptor leads to oligomerization and conformation changes in the IL-2R subunits resulting in downstream signaling starting with phosphorylation of JAK1 and JAK3 (PubMed:7973659). In turn, JAK1 and JAK3 phosphorylate the receptor to form a docking site leading to the phosphorylation of several substrates including STAT5 (PubMed:8580378). This process leads to activation of several pathways including STAT, phosphoinositide-3-kinase/PI3K and mitogen-activated protein kinase/MAPK pathways (PubMed:25142963). Functions as a T-cell growth factor and can increase NK-cell cytolytic activity as well (PubMed:6608729). Promotes strong proliferation of activated B-cells and subsequently immunoglobulin production (PubMed:6438535). Plays a pivotal role in regulating the adaptive immune system by controlling the survival and proliferation of regulatory T-cells, which are required for the maintenance of immune tolerance. Moreover, participates in the differentiation and homeostasis of effector T-cell subsets, including Th1, Th2, Th17 as well as memory CD8-positive T-cells.
IL-4	IL4	Interleukin 4	Cytokine secreted primarily by mast cells, T-cells, eosinophils, and basophils that plays a role in regulating antibody production, hematopoiesis and inflammation, and the development of effector T-cell responses (PubMed:3016727, 1993171).

			Induces the expression of class II MHC molecules on resting B-cells. Enhances both secretion and cell surface expression of IgE and IgG1 (PubMed:1993171). Regulates also the expression of the low affinity Fc receptor for IgE (CD23) on both lymphocytes and monocytes (PubMed:2521231). Positively regulates IL31RA expression in macrophages. Stimulates autophagy in dendritic cells by interfering with mTORC1 signaling and through the induction of RUFY4. In addition, plays a critical role in higher functions of the normal brain, such as memory and learning (By similarity). Upon binding to IL4, IL4R receptor dimerizes either with the common IL2R gamma chain/IL2RG to produce the type 1 signaling complex, located mainly on hematopoietic cells, or with the IL13RA1 to produce the type 2 complex, which is expressed also on nonhematopoietic cells (PubMed:10219247, 11526337, 18243101). Engagement of both types of receptors initiates JAK3 and to a lower extend JAK1 phosphorylation leading to activation of the signal transducer and activator of transcription 6/STAT6 (PubMed:7721895).
TNF-α	TNF	Tumor Necrosis Factor	Cytokine that binds to TNFRSF1A/TNFR1 and TNFRSF1B/TNFBR. It is mainly secreted by macrophages and can induce cell death of certain tumor cell lines. It is potent pyrogen causing fever by direct action or by stimulation of interleukin-1 secretion and is implicated in the induction of cachexia, Under certain conditions it can stimulate cell proliferation and induce cell differentiation. Impairs regulatory T-cells (Treg) function in individuals with rheumatoid arthritis via FOXP3 dephosphorylation. Up-regulates the expression of protein phosphatase 1 (PP1), which dephosphorylates the key 'Ser-418' residue of FOXP3, thereby inactivating FOXP3 and rendering Treg cells functionally defective (PubMed:23396208).

			Key mediator of cell death in the anticancer action of BCG-stimulated neutrophils in combination with DIABLO/SMAC mimetic in the RT4v6 bladder cancer cell line (PubMed:22517918, 16829952, 23396208).<br>Induces insulin resistance in adipocytes via inhibition of insulin-induced IRS1 tyrosine phosphorylation and insulin-induced glucose uptake.<br>Induces GKAP42 protein degradation in adipocytes which is partially responsible for TNF-induced insulin resistance (By similarity).<br>Plays a role in angiogenesis by inducing VEGF production synergistically with IL1B and IL6 (PubMed:12794819).<br>TNFA_HUMAN,P01375<br>The TNF intracellular domain (ICD) form induces IL12 production in dendritic cells.
IL-6	IL6	Interleukin 6	Cytokine with a wide variety of biological functions in immunity, tissue regeneration, and metabolism. Binds to IL6R, then the complex associates to the signaling subunit IL6ST/gp130 to trigger the intracellular IL6-signaling pathway (Probable). The interaction with the membrane-bound IL6R and IL6ST stimulates 'classic signaling', whereas the binding of IL6 and soluble IL6R to IL6ST stimulates 'trans-signaling'. Alternatively, 'cluster signaling' occurs when membrane-bound IL6:IL6R complexes on transmitter cells activate IL6ST receptors on neighboring receiver cells (Probable).<br>IL6_HUMAN,P05231 IL6 is a potent inducer of the acute phase response. Rapid production of IL6 contributes to host defense during infection and tissue injury, but excessive IL6 synthesis is involved in disease pathology.In the innate immune response, is synthesized by myeloid cells, such as macrophages and dendritic cells, upon recognition of pathogens through toll-like receptors (TLRs) at the site of infection or tissue injury (Probable). In the adaptive immune response, is required for the differentiation of B cells into immunoglobulin-secreting cells.

			Plays a major role in the differentiation of CD4(+) T cell subsets.<br>Essential factor for the development of T follicular helper (Tfh) cells that are required for the induction of germinal-center formation.<br>Required to drive naive CD4(+) T cells to the Th17 lineage.<br>Also required for proliferation of myeloma cells and the survival of plasmablast cells (By similarity). IL6_HUMAN,P05231<br>Acts as an essential factor in bone homeostasis and on vessels directly or indirectly by induction of VEGF, resulting in increased angiogenesis activity and vascular permeability (PubMed:17075861, 12794819).<br>Induces, through 'trans-signaling' and synergistically with IL1B and TNF, the production of VEGF (PubMed:12794819).<br>Involved in metabolic controls, is discharged into the bloodstream after muscle contraction increasing lipolysis and improving insulin resistance (PubMed:20823453).<br>'Trans-signaling' in central nervous system also regulates energy and glucose homeostasis (By similarity).<br>Mediates, through GLP-1, crosstalk between insulin-sensitive tissues, intestinal L cells and pancreatic islets to adapt to changes in insulin demand (By similarity).<br>Also acts as a myokine (Probable).<br>Plays a protective role during liver injury, being required for maintenance of tissue regeneration (By similarity).<br>Also has a pivotal role in iron metabolism by regulating HAMP/hepcidin expression upon inflammation or bacterial infection (PubMed:15124018).<br>Through activation of IL6ST-YAP-NOTCH pathway, induces inflammation-induced epithelial regeneration (By similarity).
IL-8	CXCL8	C-X-C Motif Chemokine Ligand 8	Chemotactic factor that mediates inflammatory response by attracting neutrophils, basophils, and T-cells to clear pathogens and protect the host from infection (PubMed:7636208, 18692776).

			Also plays an important role in neutrophil activation (PubMed:9623510, 2145175).<br>Released in response to an inflammatory stimulus, exerts its effect by binding to the G-protein-coupled receptors CXCR1 and CXCR2, primarily found in neutrophils, monocytes and endothelial cells (PubMed:1840701, 1891716).<br>G-protein heterotrimer (alpha, beta, gamma subunits) constitutively binds to CXCR1/CXCR2 receptor and activation by IL8 leads to beta and gamma subunits release from Galpha (GNAI2 in neutrophils) and activation of several downstream signaling pathways including PI3K and MAPK pathways (PubMed:8662698, 11971003).
IQCE	IQCE	IQ Motif Containing E	Component of the EvC complex that positively regulates ciliary Hedgehog (Hh) signaling (By similarity).<br>Required for proper limb morphogenesis (PubMed:28488682).
IRAK1	IRAK1	Interleukin 1 Receptor Associated Kinase 1	Serine/threonine-protein kinase that plays a critical role in initiating innate immune response against foreign pathogens.<br>Involved in Toll-like receptor (TLR) and IL-1R signaling pathways.<br>Is rapidly recruited by MYD88 to the receptor-signaling complex upon TLR activation.<br>Association with MYD88 leads to IRAK1 phosphorylation by IRAK4 and subsequent autophosphorylation and kinase activation.<br>Phosphorylates E3 ubiquitin ligases Pellino proteins (PELI1, PELI2 and PELI3) to promote pellino-mediated polyubiquitination of IRAK1.<br>Then, the ubiquitin-binding domain of IKBKG/NEMO binds to polyubiquitinated IRAK1 bringing together the IRAK1-MAP3K7/TAK1-TRAF6 complex and the NEMO-IKKA-IKKB complex.<br>In turn, MAP3K7/TAK1 activates IKKs (CHUK/IKKA and IKBKB/IKKB) leading to NF-kappa-B nuclear translocation and activation.

			Alternatively, phosphorylates TIRAP to promote its ubiquitination and subsequent degradation.<br>Phosphorylates the interferon regulatory factor 7 (IRF7) to induce its activation and translocation to the nucleus, resulting in transcriptional activation of type I IFN genes, which drive the cell in an antiviral state.<br>When sumoylated, translocates to the nucleus and phosphorylates STAT3.
ITGB2	ITGB2	Integrin Subunit Beta 2	Integrin ITGAL/ITGB2 is a receptor for ICAM1, ICAM2, ICAM3 and ICAM4.<br>Integrin ITGAL/ITGB2 is also a receptor for the secreted form of ubiquitin-like protein ISG15; the interaction is mediated by ITGAL (PubMed:29100055).<br>Integrins ITGAM/ITGB2 and ITGAX/ITGB2 are receptors for the iC3b fragment of the third complement component and for fibrinogen.<br>Integrin ITGAX/ITGB2 recognizes the sequence G-P-R in fibrinogen alpha-chain.<br>Integrin ITGAM/ITGB2 recognizes P1 and P2 peptides of fibrinogen gamma chain.<br>Integrin ITGAM/ITGB2 is also a receptor for factor X.<br>Integrin ITGAD/ITGB2 is a receptor for ICAM3 and VCAM1.<br>Contributes to natural killer cell cytotoxicity (PubMed:15356110).<br>Involved in leukocyte adhesion and transmigration of leukocytes including T-cells and neutrophils (PubMed:11812992, 28807980).<br>Triggers neutrophil transmigration during lung injury through PTK2B/PYK2-mediated activation (PubMed:18587400).<br>Integrin ITGAL/ITGB2 in association with ICAM3, contributes to apoptotic neutrophil phagocytosis by macrophages (PubMed:23775590).<br>In association with alpha subunit ITGAM/CD11b, required for CD177-PRTN3-mediated activation of TNF primed neutrophils (PubMed:21193407).

MAP3K7	MAP3K7	Mitogen-Activated Protein Kinase Kinase Kinase 7	Serine/threonine kinase which acts as an essential component of the MAP kinase signal transduction pathway.<br>Plays an important role in the cascades of cellular responses evoked by changes in the environment.<br>Mediates signal transduction of TRAF6, various cytokines including interleukin-1 (IL-1), transforming growth factor-beta (TGFB), TGFB-related factors like BMP2 and BMP4, toll-like receptors (TLR), tumor necrosis factor receptor CD40 and B-cell receptor (BCR).<br>Ceramides are also able to activate MAP3K7/TAK1.<br>Once activated, acts as an upstream activator of the MKK/JNK signal transduction cascade and the p38 MAPK signal transduction cascade through the phosphorylation and activation of several MAP kinase kinases like MAP2K1/MEK1, MAP2K3/MKK3, MAP2K6/MKK6 and MAP2K7/MKK7.<br>These MAP2Ks in turn activate p38 MAPKs, c-jun N-terminal kinases (JNKs) and I-kappa-B kinase complex (IKK).<br>Both p38 MAPK and JNK pathways control the transcription factors activator protein-1 (AP-1), while nuclear factor-kappa B is activated by IKK.<br>MAP3K7 activates also IKBKB and MAPK8/JNK1 in response to TRAF6 signaling and mediates BMP2-induced apoptosis.<br>In osmotic stress signaling, plays a major role in the activation of MAPK8/JNK1, but not that of NF-kappa-B.<br>Promotes TRIM5 capsid-specific restriction activity.<br>Phosphorylates RIPK1 at 'Ser-321' which positively regulates RIPK1 interaction with RIPK3 to promote necroptosis but negatively regulates RIPK1 kinase activity and its interaction with FADD to mediate apoptosis (By similarity). M3K7_HUMAN,O43318.
miR-147a	MIR147A	MicroRNA 147a	Diseases associated with MIR147A include Acute Promyelocytic Leukemia and Leukemia, Acute Myeloid.
miR-155-5p	MIR155	MicroRNA 155	Diseases associated with MIR155 include Diffuse Large B-Cell Lymphoma and Pancreatic Ductal Adenocarcinoma. Among its

			related pathways are Cell differentiation - expanded index and Toll-like receptor signaling pathway.
miR-182-5p	*MIR182*	MicroRNA 182	Diseases associated with MIR182 include Glioblastoma and Melanoma. Among its related pathways are miRNA regulation of p53 pathway in prostate cancer.
miR-183-5p	MIR183	MicroRNA 183	Diseases associated with MIR183 include Lung Cancer and Non-Alcoholic Fatty Liver Disease.
miR-203a-3p	MIR203A	MicroRNA 203a	Diseases associated with MIR203A include Psoriasis and Leukemia, Chronic Myeloid. Among its related pathways are Cell differentiation - expanded index and Interactions between immune cells and microRNAs in tumor microenvironment.
miR-9-5p	MIR9-1	MicroRNA 9-1	Diseases associated with MIR9-1 include Oral Squamous Cell Carcinoma and Schizophrenia. Among its related pathways are Cell differentiation - expanded index and miRNAs involvement in the immune response in sepsis.
MX1	MX1	MX Dynamin Like GTPase 1	Interferon-induced dynamin-like GTPase with antiviral activity against a wide range of RNA viruses and some DNA viruses.<br>Its target viruses include negative-stranded RNA viruses and HBV through binding and inactivation of their ribonucleocapsid.<br>May also antagonize reoviridae and asfarviridae replication.<br>Inhibits thogoto virus (THOV) replication by preventing the nuclear import of viral nucleocapsids.<br>Inhibits La Crosse virus (LACV) replication by sequestering viral nucleoprotein in perinuclear complexes, preventing genome amplification, budding, and egress.<br>Inhibits influenza A virus (IAV) replication by decreasing or delaying NP synthesis and by blocking endocytic traffic of incoming virus particles.<br>Enhances ER stress-mediated cell death after influenza virus infection.<br>May regulate the calcium channel activity of TRPCs.

MYD88	MYD88	MYD88 Innate Immune Signal Transduction Adaptor	Adapter protein involved in the Toll-like receptor and IL-1 receptor signaling pathway in the innate immune response (PubMed:15361868, 18292575, 33718825).<br>Acts via IRAK1, IRAK2, IRF7 and TRAF6, leading to NF-kappa-B activation, cytokine secretion and the inflammatory response (PubMed:15361868, 24316379, 19506249).<br>Increases IL-8 transcription (PubMed:9013863).<br>Involved in IL-18-mediated signaling pathway.<br>Activates IRF1 resulting in its rapid migration into the nucleus to mediate an efficient induction of IFN-beta, NOS2/INOS, and IL12A genes.<br>Upon TLR8 activation by GU-rich single-stranded RNA (GU-rich RNA) derived from viruses such as SARS-CoV-2, SARS-CoV and HIV-1, induces IL1B release through NLRP3 inflammasome activation (PubMed:33718825).<br>MyD88-mediated signaling in intestinal epithelial cells is crucial for maintenance of gut homeostasis and controls the expression of the antimicrobial lectin REG3G in the small intestine (By similarity).
MYOM2	MYOM2	Myomesin 2	Major component of the vertebrate myofibrillar M band.<br>Binds myosin, titin, and light meromyosin.<br>This binding is dose dependent. MYOM2_HUMAN,P54296.
NF-kappaβ	MAP3K14	Mitogen-Activated Protein Kinase Kinase Kinase 14	Lymphotoxin beta-activated kinase which seems to be exclusively involved in the activation of NF-kappa-B and its transcriptional activity. Promotes proteolytic processing of NFKB2/P100, which leads to activation of NF-kappa-B via the non-canonical pathway.<br>Could act in a receptor-selective manner.
PDE3B	PDE3B	Phosphodiesterase 3B	Cyclic nucleotide phosphodiesterase with a dual-specificity for the second messengers cAMP and cGMP, which are key regulators of many important physiological process (PubMed:14592490, 21393242).<br>Regulates angiogenesis by inhibiting the cAMP-dependent guanine nucleotide exchange factor RAPGEF3 and downstream

			phosphatidylinositol 3-kinase gamma-mediated signaling (PubMed:21393242).<br>Controls cardiac contractility by reducing cAMP concentration in cardiocytes (By similarity).
PPARA	PPARA	Peroxisome Proliferator Activated Receptor Alpha	Ligand-activated transcription factor.<br>Key regulator of lipid metabolism.<br>Activated by the endogenous ligand 1-palmitoyl-2-oleoyl-sn-glycerol-3-phosphocholine (16:0/18:1-GPC).<br>Activated by oleylethanolamide, a naturally occurring lipid that regulates satiety.<br>Receptor for peroxisome proliferators such as hypolipidemic drugs and fatty acids.<br>Regulates the peroxisomal beta-oxidation pathway of fatty acids.<br>Functions as transcription activator for the ACOX1 and P450 genes.<br>Transactivation activity requires heterodimerization with RXRA and is antagonized by NR2C2.<br>May be required for the propagation of clock information to metabolic pathways regulated by PER2. PPARA_HUMAN,Q07869.
TGFB1I1	TGFB1I1	Transforming Growth Factor Beta 1 Induced Transcript 1	Functions as a molecular adapter coordinating multiple protein-protein interactions at the focal adhesion complex and in the nucleus.<br>Links various intracellular signaling modules to plasma membrane receptors and regulates the Wnt and TGFB signaling pathways.<br>May also regulate SLC6A3 and SLC6A4 targeting to the plasma membrane hence regulating their activity.<br>In the nucleus, functions as a nuclear receptor coactivator regulating glucocorticoid, androgen, mineralocorticoid and progesterone receptor transcriptional activity.<br>May play a role in the processes of cell growth, proliferation, migration, differentiation and senescence.<br>May have a zinc-dependent DNA-binding activity.
TLR2	TLR2	Toll Like Receptor 2	Cooperates with LY96 to mediate the innate immune response to bacterial lipoproteins and other microbial cell wall components.

	Cooperates with TLR1 or TLR6 to mediate the innate immune response to bacterial lipoproteins or lipopeptides (PubMed:21078852, 17889651).<br>Acts via MYD88 and TRAF6, leading to NF-kappa-B activation, cytokine secretion and the inflammatory response.<br>May also activate immune cells and promote apoptosis in response to the lipid moiety of lipoproteins (PubMed:10426995, 10426996).<br>Recognizes mycoplasmal macrophage-activating lipopeptide-2kD (MALP-2), soluble tuberculosis factor (STF), phenol-soluble modulin (PSM) and B.burgdorferi outer surface protein A lipoprotein (OspA-L) cooperatively with TLR6 (PubMed:11441107).<br>Stimulation of monocytes in vitro with M.tuberculosis PstS1 induces p38 MAPK and ERK1/2 activation primarily via this receptor, but also partially via TLR4 (PubMed:16622205).<br>MAPK activation in response to bacterial peptidoglycan also occurs via this receptor (PubMed:16622205).<br>Acts as a receptor for M.tuberculosis lipoproteins LprA, LprG, LpqH and PstS1, some lipoproteins are dependent on other coreceptors (TLR1, CD14 and/or CD36); the lipoproteins act as agonists to modulate antigen presenting cell functions in response to the pathogen (PubMed:19362712).<br>M.tuberculosis HSP70 (dnaK) but not HSP65 (groEL-2) acts via this protein to stimulate NF-kappa-B expression (PubMed:15809303).<br>Recognizes M.tuberculosis major T-antigen EsxA (ESAT-6) which inhibits downstream MYD88-dependent signaling (shown in mouse) (By similarity).<br>Forms activation clusters composed of several receptors depending on the ligand, these clusters trigger signaling from the cell surface and subsequently are targeted to the Golgi in a lipid-raft dependent pathway.

			Forms the cluster TLR2:TLR6:CD14:CD36 in response to diacylated lipopeptides and TLR2:TLR1:CD14 in response to triacylated lipopeptides (PubMed:16880211).<br>Required for normal uptake of M.tuberculosis, a process that is inhibited by M.tuberculosis LppM (By similarity).
VNN1	VNN1	Vanin 1	Amidohydrolase that hydrolyzes specifically one of the carboamide linkages in D-pantetheine thus recycling pantothenic acid (vitamin B5) and releasing cysteamine.
XKR6	XKR6	XK Related 6	Predicted to be involved in apoptotic process involved in development; engulfment of apoptotic cell; and phosphatidylserine exposure on apoptotic cell surface. Predicted to be integral component of membrane. Predicted to be active in plasma membrane.
ZNF718	ZNF718	Zinc Finger Protein 718	May be involved in transcriptional regulation.
ZNF804A	ZNF804A	Zinc Finger Protein 804A	Diseases associated with ZNF804A include Schizophrenia and Bipolar Disorder. An important paralog of this gene is GPATCH8.
